# Metabolic labeling with an alkyne probe reveals similarities and differences in the prenylomes of several brain-derived cell lines and primary cells

**DOI:** 10.1038/s41598-021-83666-3

**Published:** 2021-02-23

**Authors:** Kiall F. Suazo, Angela Jeong, Mina Ahmadi, Caroline Brown, Wenhui Qu, Ling Li, Mark D. Distefano

**Affiliations:** 1 Department of Chemistry, University of Minnesota, Minneapolis, MN 55455 USA; 2 Department of Experimental and Clinical Pharmacology, University of Minnesota, Minneapolis, MN 55455 USA; 3 Graduate Program in Neuroscience, University of Minnesota, Minneapolis, MN 55455 USA

**Keywords:** Chemical tools, Lipids, Proteomics

## Abstract

Protein prenylation involves the attachment of one or two isoprenoid group(s) onto cysteine residues positioned near the C-terminus. This modification is essential for many signal transduction processes. In this work, the use of the probe C15AlkOPP for metabolic labeling and identification of prenylated proteins in a variety of cell lines and primary cells is explored. Using a single isoprenoid analogue, 78 prenylated protein groups from the three classes of prenylation substrates were identified including three novel prenylation substrates in a single experiment. Applying this method to three brain-related cell lines including neurons, microglia, and astrocytes showed substantial overlap (25%) in the prenylated proteins identified. In addition, some unique prenylated proteins were identified in each type. Eight proteins were observed exclusively in neurons, five were observed exclusively in astrocytes and three were observed exclusively in microglia, suggesting their unique roles in these cells. Furthermore, inhibition of farnesylation in primary astrocytes revealed the differential responses of farnesylated proteins to an FTI. Importantly, these results provide a list of 19 prenylated proteins common to all the cell lines studied here that can be monitored using the C15AlkOPP probe as well as a number of proteins that were observed in only certain cell lines. Taken together, these results suggest that this chemical proteomic approach should be useful in monitoring the levels and exploring the underlying role(s) of prenylated proteins in various diseases.

## Introduction

**Figure 1:**
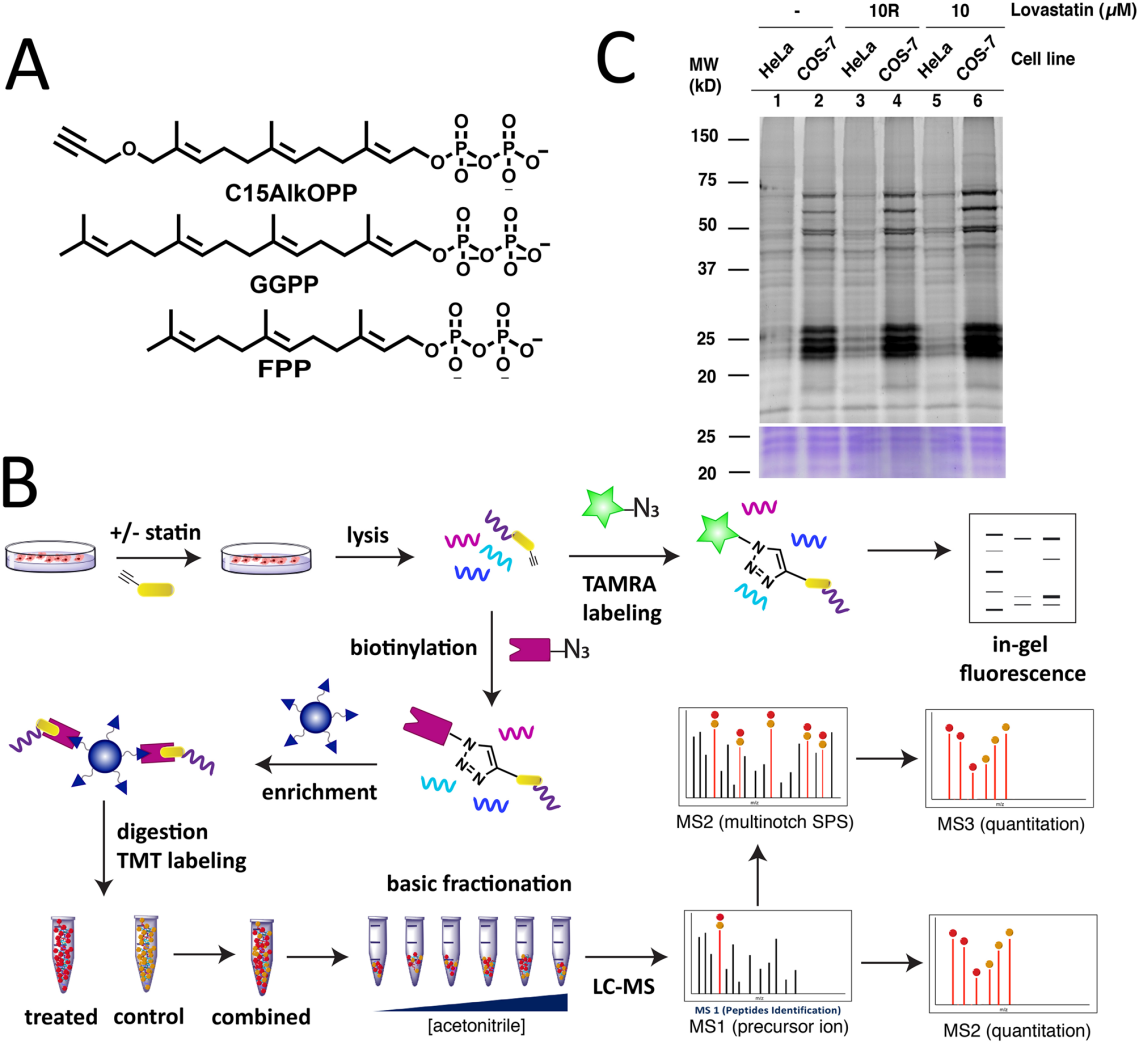
The probe C15AlkOPP allows labeling of prenylated proteins for detection and proteomic identification. **(A)** Structures of the native isoprenoids FPP and GGPP and the alkyne-modified analogue C15AlkOPP. **(B)** Scheme for metabolic labeling with C15AlkOPP in cultured cells. Labeled prenylated proteins are detected through click reaction with a fluorophore and subsequent in-gel fluorescence analysis. Prenylated proteins are enriched through conjugation with biotin and subsequent pulldown with avidin beads. Isolated proteins are digested and peptides are labeled with TMT reagent for identification and quantitation through LC–MS/MS analysis. Quantitative proteomic analysis can be performed using MS2-based or multinotch SPS-MS3 approaches. **(C)** In-gel fluorescence analysis on COS-7 and HeLa cells treated with 10 μM C15AlkOPP. COS-7 cells display superior labeling compared with HeLa cells. The presence of 10 μM lovastatin enhances probe incorporation whether it is retained (10) or removed (10R) from the culture media during metabolic labeling.

Post-translational modifications (PTMs) of proteins are important for regulation of their biological function, as well as in modulating enzymatic activities and cellular localization^1^. Among these modifications, protein prenylation is estimated to occur on 2% of the mammalian proteome, which is essential for stable anchoring of proteins to membranes, mediating protein–protein interactions, and protein trafficking^2^. Protein prenylation is the irreversible attachment of a farnesyl or geranylgeranyl group(s) onto a cysteine residue near the C-terminus of a protein. Farnesyltransferase (FTase) and geranylgeranyltransferase type 1 (GGTase-I) recognize the conventional CaaX-box prenylation motif (C = cysteine, a and X = any amino acid) and append a single isoprenoid from farnesyl diphosphate (FPP) and geranylgeranyl diphosphate (GGPP), respectively (Fig. 1A)^3,4^. Interestingly, recent studies have shown that extended C(X)_3_X motifs in model proteins can be farnesylated within cells^5^, while shortened Cxx peptides are also acceptable FTase substrates in vitro^6^. Dual geranylgeranylation on a family of Rab proteins also occurs on C-terminal CCXX, CXC, or CC motifs catalyzed by rab geranylgeranyltransferase (GGTase-II or RabGGTase)^7^. Recently, a new GGTase (GGTase-III) has been identified with FBXL2 and Ykt6 as its only known substrates identified thus far^8,9^.

Inhibiting protein prenylation using prenyltransferase inhibitors (PTIs) has been of interest in clinical studies for potential anticancer therapies. In particular, inhibition of farnesylation using farnesyltransferase inhibitors (FTIs) of Ras protein and its oncogenic variants that drive tumor growth was initially the main focus of those studies. While these inhibitors failed in early clinical trials, they show great promise in precision medicine applications particularly when cancers can be linked to specific H-Ras mutations. A Phase 2 clinical trial using the FTI Tipifarnib for H-Ras-driven head and neck cancer is currently in progress^10^. The inhibitory effects of FTIs have also been found to be potentially useful for therapeutic applications in other diseases and pathologies where prenylation is required. These include neurodegenerative diseases, progeria, bacterial, viral, and protozoal infections^11,12^. Very recently, the use of the FTI Lonafarnib to inhibit the farnesylation of nuclear lamin was approved by the FDA for the treatment of Hutchinson-Gilford progeria syndrome^13^. Overall, these studies have made it clear that a better understanding of which proteins are actually prenylated is required to develop more effective therapeutic strategies.

Earlier methods to identify prenylated proteins relied on autoradiography techniques where tritiated forms of mevalonic acid, a precursor in isoprenoid biosynthesis, as well as [^3^H]FPP and [^3^H]GGPP were used to label prenylated proteins^14–16^. However, these methods are laborious, expensive, less sensitive, and often require longer periods of exposure time. Immunological methods using isoprenoid-directed antibodies have not proved to be generally useful for prenylated protein identification and have only been used in a limited number of cases^17,18^. Related work with antibodies that target an aniline-containing analogue of FPP successfully identified 22 prenylated proteins^19^. Mass spectrometry-based chemical proteomics has emerged as a powerful tool to identify proteins with specific PTMs. For labeling of prenylated proteins, isoprenoid probes with small biorthogonal functionalities including azides or alkynes are metabolically introduced into cells of interest^20–24^. The cellular machinery incorporates these probes into the prenylated proteins in lieu of the native isoprenoids. Subsequent tagging through click reaction with a fluorophore or a biotin handle allows for selective detection and enrichment, enabling identification and quantitation of the prenylated proteins in a high throughput fashion. Initial efforts employed azide-functionalized isoprenoids^20,25^ but in recent years a variety of alkyne-containing analogues have been prepared to capitalize on the lower background obtainable with those types of probes^26,27^.

In this work, the use of the probe C15AlkOPP for metabolic labeling and identification of prenylated proteins in a variety of cell lines and primary cells is explored. First experiments in COS-7 cells were performed to determine how many prenylated proteins could be identified. Efforts to correlate those results with data reflecting substrate efficiency and protein expression levels are described. The result of using either MS2 or MS3 approaches for proteomic analysis is reported. Results from the COS-7 experiments were compared with similar data from HeLa cells to examine how the enrichable prenylated proteins vary between cell lines. Finally, analysis of the prenylomes from three different brain-derived cell lines and primary astrocytes was performed to obtain a list of prenylated proteins whose levels can be quantified to study dysregulation of protein prenylation in brain-related diseases.

## Results

### Incorporation via metabolic labeling of C15AlkOPP varies in different cell lines

The probe C15AlkOPP bearing an alkyne functional group (Fig. 1A) has previously been shown to be efficiently incorporated via metabolic labeling into all three classes of prenylated proteins in a number of different individual cell lines^27–29^. However, to date, only limited comparisons between different cell lines have been made making it difficult to assess the generality of this approach^27,30,31^. To make that type of comparison here, the success of probe incorporation was first evaluated through in-gel fluorescence analysis. Cells were incubated with the probe, lysed and subjected to click reaction with TAMRA-N_3_ through copper-catalyzed cycloaddition (Fig. 1B). Labeled proteins were resolved via SDS-PAGE and TAMRA fluorescence resulting from labeling of the prenylated proteins was detected. We have previously shown that using the free alcohol form of the probe (C15AlkOH), which is metabolically converted into its diphosphate form (the bonafide substrate for protein prenylation) results in less efficient labeling compared to incubation with the phosphorylated form (C15AlkOPP) in COS-7 cells based on in-gel fluorescence analysis^29^. Hence, efforts here focused on experiments with the diphosphate form. Incubation of cells with 10 μM C15AlkOPP for 24 h is sufficient to achieve maximum label incorporation; while labeling can be increased using higher probe concentration, that increase is offset by greater background labeling.

Enhancing the labeling of prenylated proteins can often be achieved through suppression of the native production of FPP and GGPP using various statins or fosmidomycin in organisms that lack the mevalonate pathway^28,32^. In general, it is important to optimize the statin concentration used since there is a balance between improving label incorporation and cellular toxicity due to the statin. Moreover, various cell lines may respond differently to statins^33^ and optimization of statin treatment may be required for effective labeling of prenylated proteins in a given cell line. Previous results with COS-7 and HeLa cells showed that 10 µM lovastatin augments label incorporation with no apparent toxicity^32^; hence, that concentration was used throughout the experiments reported here. Using COS-7 cells, some enhancement of labeling was observed in the 25, 50, and 75 kDa regions in cells pre-incubated with 10 μM lovastatin for 6 h followed by probe treatment (Fig. 1c, lane 6) compared to without lovastatin treatment (Fig. 1C, lane 2); however, in this cell line, that increase was relatively modest. Interestingly, we have observed that lovastatin induces some level of toxicity in cells when left in the culture media for longer periods of time. Accordingly, we evaluated the effect of removing the lovastatin after pre-incubation prior to probe treatment (Fig. 1C, lane 4). This resulted in only a minor decrease in labeling compared with that obtained when lovastatin remained present during metabolic labeling. Overall, the effects of lovastatin on metabolic labeling in COS-7 cells are modest.

In contrast, different results were obtained in HeLa cells. In a side-by-side comparison of metabolic labeling in COS-7 and HeLa cells in the presence (Fig. 1C lanes 3 and 5) or absence (Fig. 1C, lane 1) of lovastatin, there is clearly less overall labeling in HeLa cells. Regardless of whether the lovastatin is retained or removed in the culture media, it does not increase labeling in HeLa cells to the level observed in COS-7. However, the presence of the statin in HeLa cells does modestly increase the level of probe incorporation relative to that observed in the absence of the statin (compare lane 1 with lanes 3 and 5 particularly in the 25 kDa region). Additionally, statin removal prior to metabolic labeling does appear to alter the pattern of bands observed in the 25 kDa region with fewer labeled bands being seen when the statin remained present during metabolic labeling. This is consistent with previous results reported with HeLa cells and may reflect the effects of statin toxicity or a different pleiotrophic mechanism. Given the goal of identifying a maximal set of prenylated proteins (prenylome) in a mammalian cell line, COS-7 was chosen for subsequent prenylomic analysis due to its superior labeling with C15AlkOPP.

### Quantitative prenylomic analysis using COS-7 cells identifies approximately 80 different protein groups

To perform the prenylomic analysis (Fig. 1B), COS-7 cells were pre-treated with lovastatin followed by the addition of C15AlkOPP or FPP as a control and incubated for another 24 h with lovastatin retained in the media. FPP was included in the control (instead of vehicle alone), to offset any potential physiological effect of added isoprenoid diphosphate and to reverse perturbations caused by the presence of the statin. Labeled protein lysates from triplicate samples were subjected to click reaction with biotin-N_3_ to selectively enrich the labeled prenylated proteins through incubation with avidin beads. The beads were washed under stringent conditions to remove non-specifically bound proteins and the resulting immobilized proteins were digested on-bead using trypsin. Equal amounts of the collected tryptic peptides were subjected to TMT-labeling and combined. Samples were fractionated under high pH reversed-phase conditions at different acetonitrile concentrations to decrease the sample complexity. Each fraction was then analyzed via LC–MS followed by database searching and statistical analysis to identify the enriched prenylated proteins. Since the prenylated peptides remain bound to the avidin resin after proteolysis, protein identification was based on the enrichment and identification of other peptides derived from individual proteins. Efforts to identify proteins based on specific prenylated peptides have not been generally successful although several methods to accomplish that are currently being explored^31,34^.

**Figure 2:**
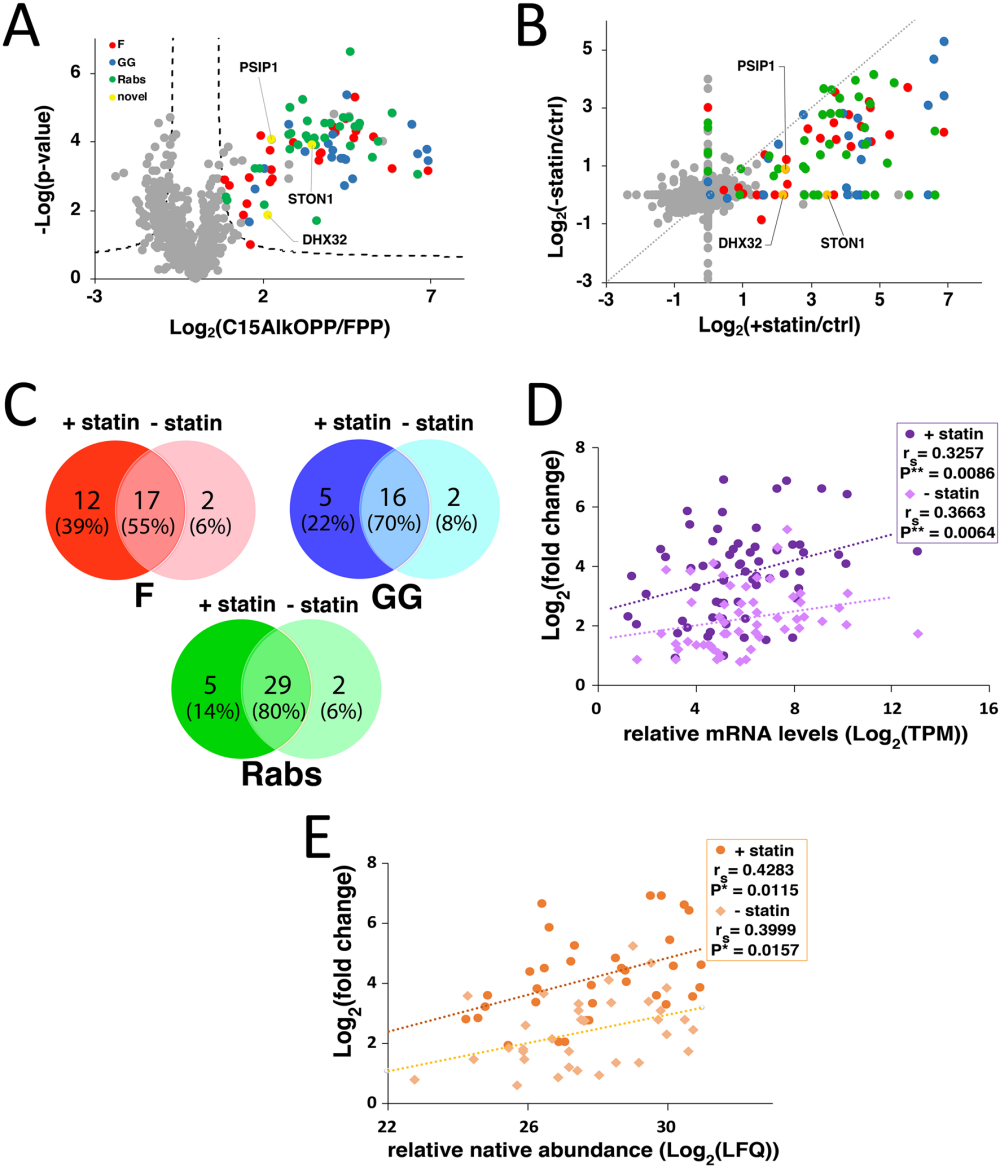
A higher number of prenylated proteins were identified when metabolic labeling was performed in the presence of lovastatin. **(A)** A volcano plot (FDR = 0.01, s0 = 0.5) showing the statistically enriched prenylated proteins identified in COS-7 cells from triplicate samples (red : farnesylated; blue : geranylgeranylated; green : rab proteins). **(B)** Fold-change correlation plot between the prenylated proteins identified in the presence or absence of lovastatin. **(C)** Venn diagrams displaying the number of proteins from the three classes of prenyltransferase substrates identified in the presence or absence of lovastatin. Protein groups were separated into individual proteins. **(D** and **E)** Correlation between the enrichment fold-change and relative mRNA levels **(D)** or native abundances **(E)** of GGTase-I substrates and Rab proteins identified in the presence or absence of lovastatin. A significant positive correlation (*p* < 0.05) determined by Spearman’s rank-order correlation was observed in the presence or absence of lovastatin.

Initially, a multinotch SPS-MS3 approach was employed to limit ion interference and achieve more accurate and precise quantitation of the detected peptides across a 2-h LC gradient^35^. In this approach, the precursor ions from MS1 are fragmented in MS2 and synchronously selected precursors (SPS) from MS2 are further fragmented at the MS3 level where the reporter ions are detected. Using this method, 78 protein groups containing putative prenylation motifs were identified to be statistically enriched from the C15AlkOPP-treated samples (Fig. 2A, Supplementary Table S3). Most of these proteins are currently annotated or were reported to be prenylated in previous prenylomic studies^27,31,36^. In enriching prenylated proteins, previous studies using a similar approach identified other proteins that do not bear prenylation motifs^31,36^. These can be proteins that are known to interact with the prenylated proteins that survive the enrichment step after washing. In our analysis, in addition to the 78 protein groups mentioned above, we also enriched five proteins known to interact with prenylated proteins: PIK3C2A, CD9, and UBA52 (interact with Rabs), KRIT1 (interacts with Rho GTPases), and ASPM (interacts with farnesylated proteins CENPF and LMNB1). This indicates that in the approach described, a small number of proteins obtained from the analysis (6%) are not prenylated proteins but are known interacting partners. In the absence of statin, a related set of 59 protein groups of statistically enriched prenylated proteins were identified (Supplementary Table S4). The vast majority of the prenylated proteins detected in the absence of statin were also observed in the presence of statin although there were three protein groups (RRAS2, RAB12, RAB43) that were unique to samples that were not pretreated with statin. Given the results from gel-based experiments showing that lovastatin enhances probe incorporation, it was not surprising that the enrichment fold-changes of the prenylated proteins were indeed higher when the cells were pre-incubated with lovastatin (Fig. 2B). Interestingly, it was found that enhanced labeling in the presence of statin was more pronounced for farnesylated proteins compared to geranylgeranylated type-I substrates and Rab proteins (Fig. 2C).

An important question concerning this experimental approach is what parameters control the enrichment and hence detection of prenylated proteins in metabolic labeling experiments? In an effort to determine the factors that influence the extent of enrichment for individual prenylated proteins, the correlation between the enrichment fold-changes and several parameters reported in the literature were explored. Such factors describe the propensity of these prenylated proteins to serve as substrates for prenylation. To account for the abundance of each prenylated protein, the mRNA levels of proteins (expressed as transcripts per million reads, TPM) were measured in COS-7 using next-generation sequencing. The values were used to normalize the enrichment fold changes of each prenylated protein and the resulting products were log-transformed. The relationship between enrichment fold-changes of farnesylated proteins and their predicted farnesylation scores (based on their C-terminal CaaX-box motifs) using two different methods was first investigated. In one analysis, the farnesylation scores obtained from Prenylation Prediction Suite (PrePS)^37^, an online web-based tool containing a database of prenylated proteins across sequenced genomes, were plotted against the normalized fold changes of the farnesylated proteins identified (Fig. S1A). Another set of predicted farnesylation scores derived from a structure-based scheme for prediction of peptide binding (FlexPepBind) developed by Furman and coworkers^38^ was used to assess their correlation with the enrichment fold-changes (Fig. S1B). In both analyses, no apparent relationship was observed between the extent of enrichment of farnesylated proteins and their predicted probability as efficient substrates for farnesylation, either in the presence or absence of lovastatin. For GGTase-I and Rab substrates, the reported relative efficiency of geranylgeranylation of their C-terminal peptides^39^ and the percent Rab prenylation efficiencies^40^ determined in vitro, respectively, were used (Fig. S1C and S1D). In the case of Rab proteins, the normalized fold change values of those that contain two prenylatable C-terminal cysteines were divided by 2. Again, no clear correlation between the extent of enrichment of individual prenylated proteins and their measured prenylation efficiencies was observed. Intriguingly, proteins sharing the same C-terminal CaaX-box motifs exhibited notable differences in enrichment including PALM and LMNA (CSIM), NAP1L1 and NAP1L4 (CKQQ), CDC42 and RRAS (CVLL), and RAP1A and RAC1 (CLLL) (Fig. S1A, S1C). Importantly, LMNA is derived from the maturation of prelamin A, where the farnesylated C-terminal peptide is cleaved off^41^. The loss of the prenylated peptide will impact the native abundance of LMNA available for enrichment, as only the premature LMNA bears the tagged isoprenoid analogue. Such additional processing clearly complicates subsequent analysis.

A large fraction of prenylated proteins belong to the family of small GTPases that have molecular weights of approximately 25 kDa. This is strikingly illustrated by the intense labeling observed in the 25 kDa region of gels analyzed via in-gel fluorescence (Fig. 1B). To investigate a possible correlation between the extent of enrichment in a prenylomic metabolic labeling experiment and protein abundance, a quantitative proteomic analysis of the 25 kDa region was performed. Lysates from COS-7 cells cultured in the presence or absence of 10 μM lovastatin were resolved via SDS-PAGE and the proteins in the 20 to 30 kDa region were excised and prepared for label-free quantitative proteomic analysis. The relative native abundances from triplicate samples were measured as label-free quantitation (LFQ) intensities and were used to normalize the enrichment fold-changes. There was no correlation observed between the normalized fold enrichment fold changes and Rab prenylation efficiency (Fig. S1E).

We next sought to determine whether protein abundances influence the enrichment fold-changes. Instead of using the mRNA levels or native abundances for normalization, we investigated the direct dependence of the observed enrichment fold-changes on these measures of relative protein abundance. Interestingly, a statistically significant dependence of the enrichment fold-changes with their respective expression levels and native abundances in the presence or absence of lovastatin was observed (Fig. 2D and E). This observation suggests that the enrichment of prenylated proteins obtained using metabolic labeling may be correlated with their inherent expression levels and native abundances under the conditions the probe labeling is performed. Exogenous treatment with isoprenoids or statins is known to alter expression levels of proteins, including some members of the Ras and Rho proteins that are prenylation substrates^42,43^. We compared the native abundance of the prenylated proteins in the 25 kDa region in COS-7 cells in the presence or absence of lovastatin (Fig. S2). The abundances of most of these proteins did not significantly change in the presence of lovastatin except for RhoB. Interestingly, that protein was previously reported to be sensitive to statin treatment, however, exogenous supplementation of isoprenoids can reverse the upregulation of Rhob induced by lovastatin^42,44^. As commented above, it is important to note that the control samples used in the profiling experiments described here were also treated with lovastatin and FPP, and hence dysregulatory effects of statins and isoprenoid diphosphates on expression levels of prenylated proteins are accounted for when the enrichment fold changes are calculated to determine the statistically enriched prenylated proteins in the C15AlkOPP-treated samples.

**Figure 3:**
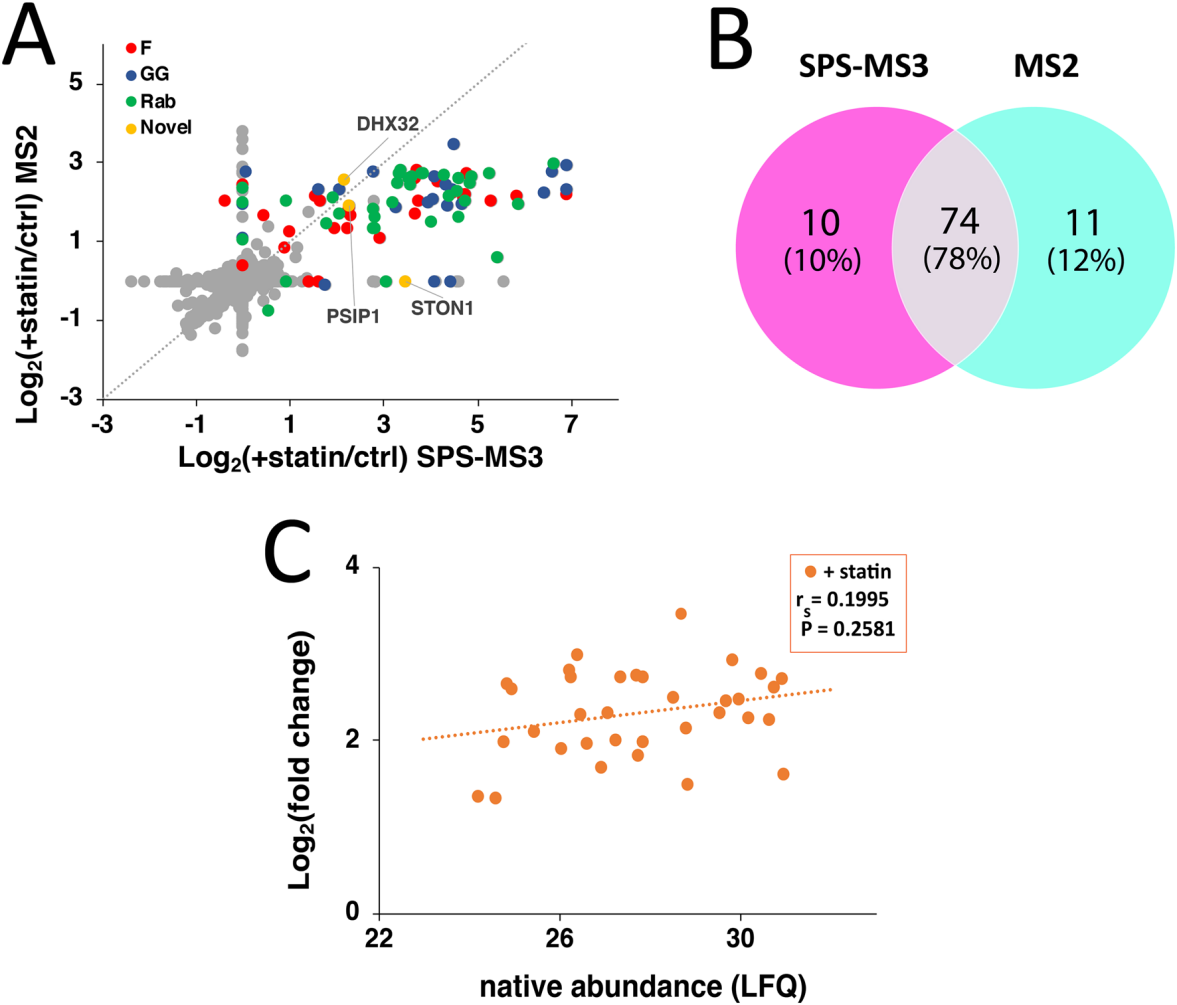
Comparison of the prenylated proteins identified from multinotch SPS-MS3 and MS2-based approaches. **(A)** Fold-change correlation plot of the prenylated proteins identified from multinotch versus MS2-level quantitative proteomic analyses. A wider dynamic range was observed employing the multinotch approach. **(B)** Summary of the number of individual prenylated proteins identified from the two approaches. Protein groups were separated into individual proteins. A similar number of proteins were identified with good overlap. **(C)** Correlation between the enrichment fold-change and relative native abundances of GGTase-I substrates and Rab proteins identified from MS2-based analysis on COS-7 in the presence of statin. No statistically significant correlation was observed.

While the multinotch SPS-MS3 approach employed on isobarically tagged peptide samples provides a more accurate quantitation of the identified proteins, the current instruments equipped with this capability are more expensive and hence often less accessible. Simpler but less sensitive orbitrap mass spectrometers capable of MS2-based identification and quantitation of proteins from TMT-labeled peptides are often more readily available. They should suffice for proteomic profiling studies when quantitative accuracy is not required. Accordingly, the samples described above were analyzed using a less sensitive orbitrap instrument and the reporter ions at the MS2 level were used to quantify the identified prenylated proteins. A total of 79 protein groups of putative prenylated proteins were identified in the presence of lovastatin (Supplementary Table S5). For most prenylated proteins identified, the enrichment fold-changes were higher in the multinotch SPS-MS3 approach compared to the MS2-based analysis (Fig. 3A). The wider dynamic range of enrichment fold-change values obtained from the SPS-MS3 approach illustrates its superior quantitative accuracy over the MS2 approach. Nevertheless, while differences between the two data sets were observed, 80% of the prenylated proteins were detected using either of the MS approaches (Fig. 3B). Such variation is common in proteomic experiments using different LC–MS instruments^45^. We further examined if the enrichment fold-changes of the identified small GTPases and Rabs correlate with their relative native abundances in COS-7 treated with lovastatin (Fig. 3C). In contrast to the SPS-MS3 approach, we did not observe a correlation of the Log_2_(fold changes) obtained from our MS2 approach with their relative native abundances. Thus, it appears that the lesser quantitative accuracy of the MS2-based analysis using the less expensive orbitrap instrument may provide less accurate data for the extent of enrichment and therefore complicates efforts to observe a possible correlation between enrichment and native abundance.

### The prenylated proteins identified in HeLa cells are a subset of those observed in COS-7 cells

**Figure 4:**
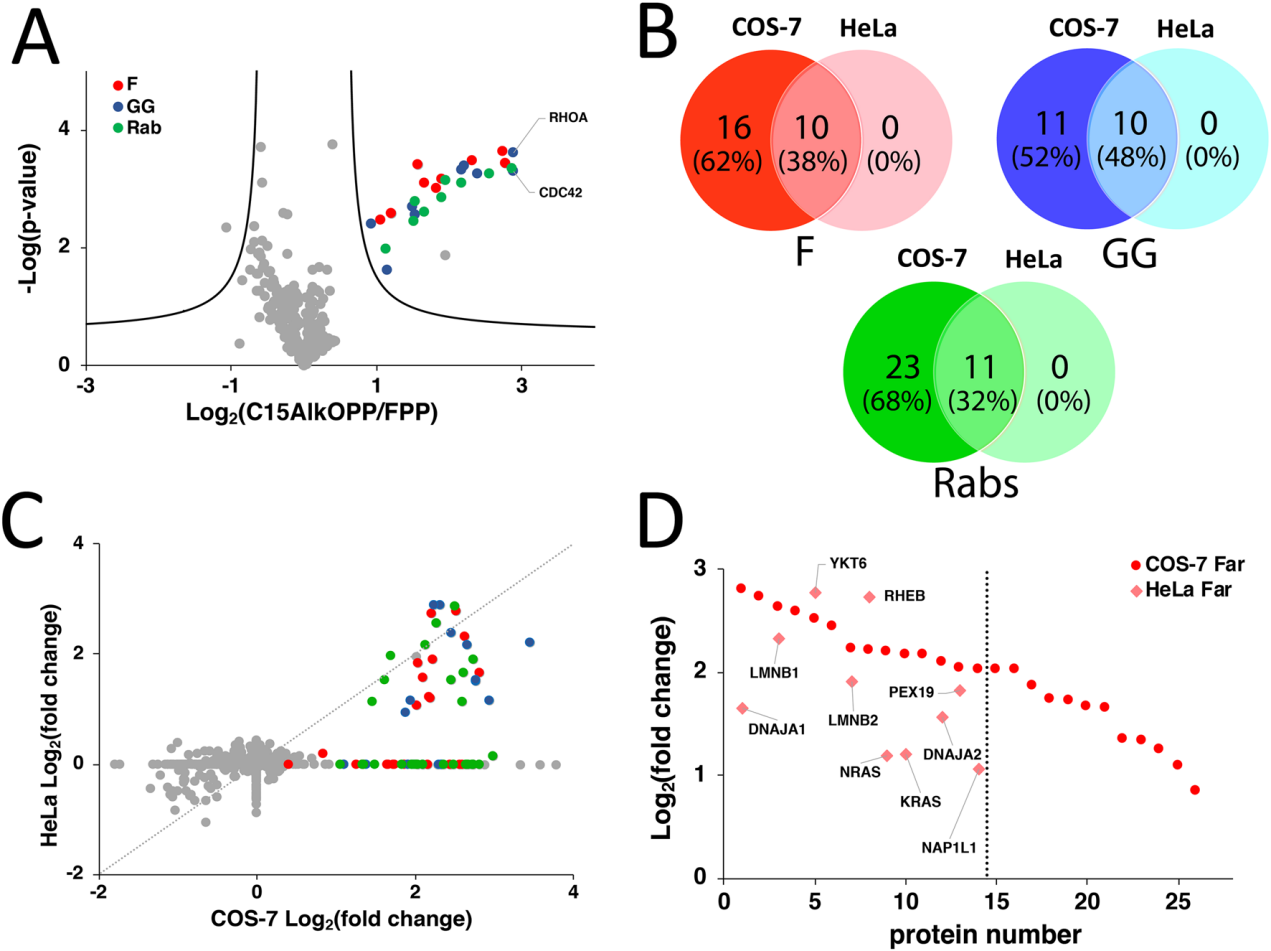
The prenylated proteins identified in HeLa is a subset of those in COS-7. **(A)** A volcano plot (FDR = 0.01, s0 = 0.5) showing the statistically enriched prenylated proteins identified in HeLa cells from triplicate samples (red : farnesylated; blue : geranylgeranylated; green : rab proteins). **(B)** Venn diagrams displaying the overlap of proteins categorized into the three classes of prenyltransferase substrates identified in COS-7 and HeLa. Proteins groups were separated into individual proteins. **(C)** Fold-change correlation plot between the prenylated proteins identified in COS-7 and HeLa. **(D)** Farnesylated proteins identified in HeLa were the most enriched prenylated proteins in COS-7 identified from the MS2-based analysis.

In the fluorescent gel-based experiments described above, the labeling efficiency in HeLa cells using the C15AlkOPP probe was significantly less than that in COS-7 cells (Fig. 1B). Albeit less pronounced, the banding pattern observed in the 25, 50, and 75 kDa regions in these two cell lines are similar. We speculated that this less efficient labeling in HeLa cells might only result in lower enrichment fold-changes but would not affect the number of prenylated proteins that could be enriched. Therefore, a proteomic analysis of the prenylated proteins in HeLa cells treated with C15AlkOPP in the presence of lovastatin using a MS2-level approach was performed. Although less accurate, this should suffice for profiling to provide the identities of the prenylated proteins labeled in cells. A total of 28 prenylated protein groups were statistically enriched in HeLa cells (Fig. 4A, Supplementary Table S6), approximately one-third of those profiled from COS-7 cells using the same LC–MS method and instrument. When the identities of these prenylated proteins categorized into the three classes of prenylation substrates were compared, we found that across all three classes, the set of prenylated proteins identified in HeLa is a subset of those identified in COS-7 cells (Fig. 4B). These were equally divided among the three classes and corresponded to less than 50% of those identified in COS-7. Furthermore, the prenylated proteins in HeLa generally possessed lower enrichment fold-changes compared to those from COS-7 cells (Fig. 4C). Therefore, the reduced labeling efficiency in HeLa not only results in diminished enrichment fold-changes but also impacted the number of enriched prenylated proteins detected.

Since a lower number of prenylated proteins were identified in HeLa cells, we speculated that these might correspond to the most efficiently labeled proteins in COS-7. Accordingly, the enrichment fold-changes of the prenylated proteins identified in both HeLa and COS-7 cells in each class of prenylation substrates were determined. Those identified in COS-7 were ranked in decreasing order of enrichment fold-change and plotted along with the corresponding prenylated proteins identified in HeLa. For both GGTase-I substrates and Rabs, the prenylated proteins identified in HeLa were distributed across the range from the most to least enriched prenylated proteins in COS-7 cells, showing no apparent relationship (Fig. S3A and 3B). Interestingly, the farnesylated proteins identified in HeLa are among the most highly enriched farnesylated proteins in COS-7 (Fig. 4D). This set of farnesylated proteins was also readily identified in COS-7 without statin treatment from the prenylomic analysis performed using SPS-MS3, which generally exhibited enhanced enrichment fold-changes in the presence of lovastatin. These proteins are perhaps the most efficiently farnesylated inside cells. To better understand this, we examined whether the C-terminal CaaX-box motifs of these farnesylated proteins in HeLa cells have predicted higher farnesylation scores (Fig. S3C). While most of these proteins have scores that predict high or medium propensity for farnesylation, NRas (CVVM) and NAP1L1 (CKQQ) manifesting low farnesylation scores were also identified.

As noted above, an apparent correlation was observed between the enrichment fold-changes and native abundances of GGTase-I substrates and Rabs in COS-7 from the SPS-MS3 approach but not in MS2-based analysis. We examined if the enrichment fold-changes of the prenylome identified in HeLa from our MS2-based analysis correlates with their relative native abundances Fig. S3D). Unfortunately, no significant correlation was observed, which may be attributed to the lower quantitative accuracy and dynamic range of MS2-based analysis in the less sophisticated orbitrap instrument. However, we did find that most proteins profiled in HeLa are those that have relatively high abundance in COS-7 (Fig. S3E). This comparison suggests that the native abundance of prenylated proteins may contribute significantly to their extent of enrichment and ability to be enriched, rather than their respective catalytic efficiencies.

### Prenylomic profiling of brain cell lines

**Figure 5:**
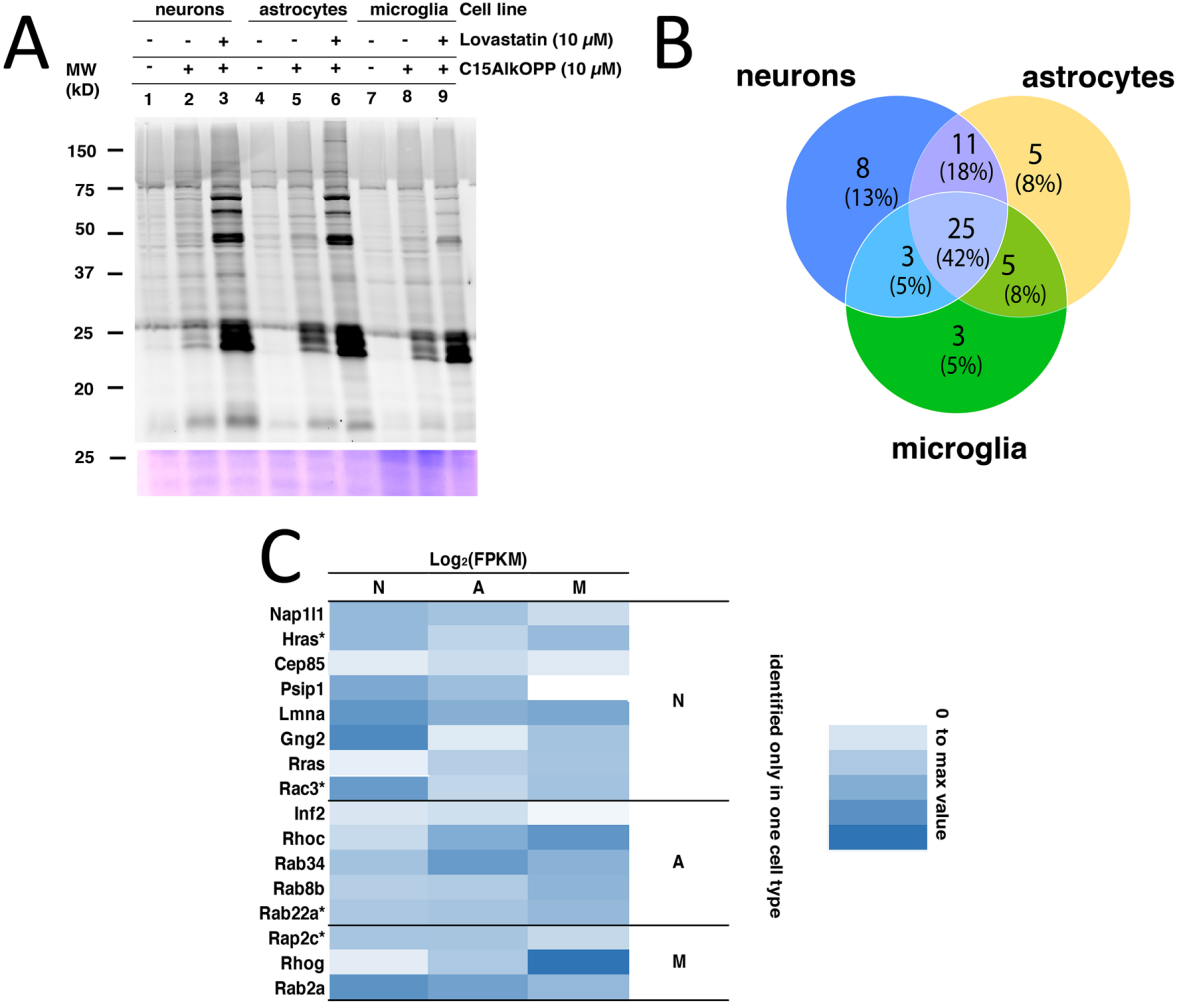
Similarities and differences in the sets of prenylated proteins identified from three different brain cell lines and primary astrocytes. **(A)** In-gel fluorescence analysis on neurons, microglia, and astrocytes treated with 10 $\mu$M C15AlkOPP in the presence or absence of 10 μM lovastatin. **(B)** Summary of the number of identified prenylated proteins that were unique or shared among neuronal cells (N), immortalized astrocytes (A), and microglial cells (M). **(C)** A heat map of the relative mRNA expression levels of the prenylated proteins uniquely identified in each cell type. *Proteins that were identified from a protein group.

The data presented above demonstrates that approximately 80 prenylated protein groups can be identified using this method in COS-7 cells. Given the aforementioned observations concerning HeLa cells, it is likely that the number and prevalence of prenylated proteins will vary between different cell lines. To study this in more detail, the differences between several closely related cell lines were investigated. In the context of the brain, stable cell lines representing the most common cell types are readily available and therefore suitable for metabolic labeling and in vitro analysis. Hence, we hypothesized that it should be possible to identify prenylated proteins that are both common between different cell types as well as reveal others that are differentially expressed or prenylated, which may contribute to the unique function of each cell type. Accordingly, neuronal cells (N2a), immortalized astrocytes, and microglial cells (BV2) were metabolically labeled with the C15AlkOPP probe in the presence or absence of lovastatin. The lysates obtained were subjected to click reaction with TAMRA-N_3_ and in-gel fluorescence analysis (Fig. 5A). In the absence of lovastatin, appreciable labeling was achieved across all cell lines (lanes 2, 5, and 8). The presence of lovastatin significantly enhanced the labeling of these bands, with some new bands appearing near the 50 and 75 kDa region (lanes 3, 6, and 9). These differential banding patterns observed between cells treated or not treated with lovastatin indicate that there are proteins that can only be detected in the presence of lovastatin. Despite the appearance of new bands in microglia, the labeling enhancement was less compared to those observed in neurons and astrocytes. It is also important to note that in the gel image, the contrast was adjusted in order for the differential banding pattern to be visualized. All brain-derived cell lines have less labeling compared to COS-7 in our in-gel fluorescence experiments.

Next, proteomic analysis of the enriched prenylated proteins within the three brain cell types in the presence of lovastatin was carried out. An MS2-based approach was employed to identify statistically enriched proteins since it suffices for profiling purposes. The total number of statistically enriched prenylated proteins in each cell line was less than that obtained with COS-7 cells, indicating that prenylome labeling was less efficient across all three brain cell lines. There were also no novel prenylated proteins detected. Among the individual prenylated proteins detected, 25 were shared (42%) by all three, with some shared by only two, or uniquely detected in one cell type (Fig. 5B). More prenylated proteins were identified from neurons with concurrent stronger background labeling (unprenylated proteins appearing to be enriched, see Fig. S4), reflecting the observed differences in labeling from the in-gel fluorescence analysis. Microglia afforded the smallest number of prenylated proteins profiled.

As discussed above, the resulting similarities and differences in the set of prenylated proteins identified may reflect their endogenous expression levels in each cell type. However, there are no available data on the native abundances of these proteins in each cell line. We therefore turned to published RNA-Seq data reporting on the mRNA expression levels of individual prenylated proteins identified in each cell type using a web-based tool (www.brainrnaseq.org)^46^. The Fragments per Kilobase of transcript per Million mapped reads (FPKM) values of genes corresponding to identified prenylated proteins were extracted from each cell type and log2 transformed. A heat map was generated to compare expression levels of prenylated proteins shared by all three cell types, showing that they were generally expressed at higher levels in microglia (Fig. S5A). The proteins identified only in both neurons and astrocytes were dominated by farnesylated proteins (Fig. S5B). There were no significant correlations between the enrichment fold-changes and the mRNA levels of the identified prenylated proteins across all cell lines. Nevertheless, CDC42 and RhoA were identified as two of the most enriched prenylated proteins with relatively high mRNA levels in all cell lines (Fig. S6).

Proteins that were uniquely identified from each cell type were of particular interest (Fig. 5C). However, some of these proteins were grouped with proteins that were also identified in other cell lines due to extensive sequence homology and may not be truly uniquely identified. Those that were identified only in neurons were mostly expressed at higher levels in neurons. In contrast, the prenylated proteins uniquely identified in astrocytes or microglia do not generally correlate with their relative expression levels, except for RhoC that is highly expressed and was detected in microglia. Overall, there was no general correlation between the expression levels of individual prenylated proteins (based on the aforementioned RNA-Seq data) and their ability to be enriched in a prenylomic profiling experiment. The relative expression levels among the different prenyltransferase enzymes, which may reflect the innate efficiency of prenylation activity in each cell type (Fig. S5C) were also examined. The catalytic subunits FntB (FTase), Pggt1B (GGTase-I), and RabggtB (RabGGTase) are generally expressed at lower levels in microglia. This may explain why fewer proteins were enriched from microglia and why those that were observed were often those with relatively higher expression levels.

### Inhibition of prenylation

As mentioned previously, the initial development of PTIs was driven by their potential pharmacological benefits in targeting oncogenic isoforms of proteins that promote tumorigenesis. However, their efficacies maybe negated owing to the ability of the key oncogenic targets to be alternatively geranylgeranylated in lieu of being farnesylated^31^. To determine the proteins displaying the switch-like behavior, a previous report on isoprenoid probe labeling in the presence of farnesyltransferase inhibitors (FTIs) showed that KRAS, NRAS, and RRAS2 can be geranylgeranylated when FTase is inhibited, indicating that these proteins may be responsible for the observed failure of FTIs in clinical trials, a result consistent with previous reports^31^. However, that experiment was performed in a human endothelial cell line.

In order to investigate the differential responses of cell lines to an FTI, COS-7 and HeLa cells were evaluated for their prenylome labeling upon FTI treatment (Fig. S7A). In COS-7 cells, the bands near the 50 and 75 kDa regions displayed significant reduction in labeling in the presence of tipifarnib, a validated and commonly used FTI. In HeLa cells, however, the low efficiency of probe incorporation makes it challenging to assess its sensitivity to FTI inhibition. Only one band near the 75 kDa region appears to be inhibited by tipifarnib. The inhibition profiles for the brain-derived cell lines also behaved similarly with that observed in HeLa in the absence of lovastatin (Fig. S7B), which can be attributed to the inherently lower probe incorporation in these cell lines compared to COS-7. Since farnesylated proteins are dependent on lovastatin treatment to enhance their probe incorporation as observed in Fig. 2C, we included lovastatin treatment to effectively label farnesylated proteins and assess the effect of FTI-induced inhibition. Indeed, the enhanced labeling induced by lovastatin clearly facilitates the observation of inhibition by tipifarnib. The strongly labeled bands near the 50 and 75 kDa regions were completely abolished in all brain-derived cell lines.

**Figure 6:**
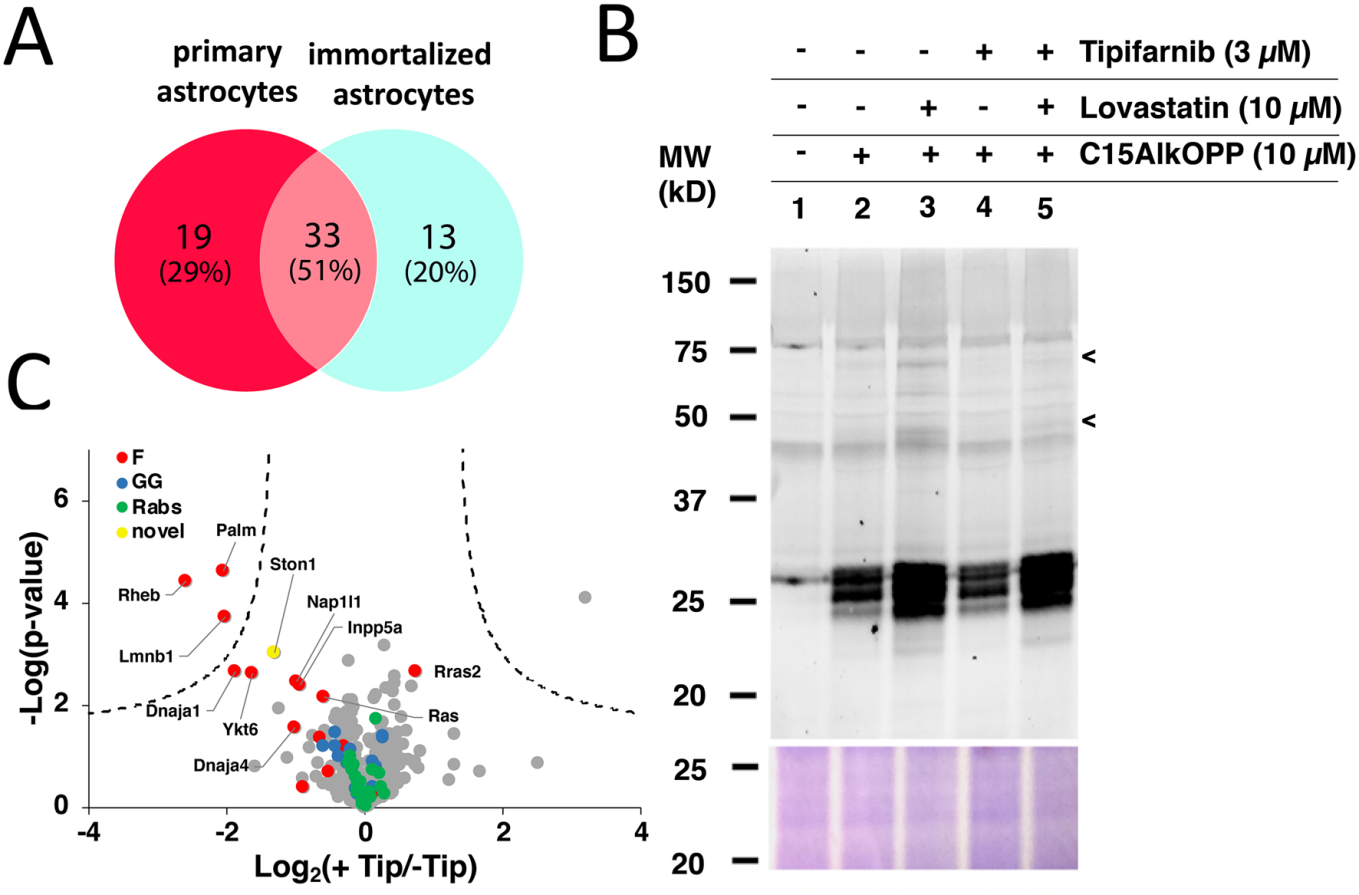
Inhibition of farnesylation in primary astrocytes. **(A)** Summary of the number of prenylated proteins identified from immortalized and primary astrocytes. **(B)** In-gel fluorescence analysis of primary astrocytes in the presence or absence of lovastatin and tipifarnib. **(C)** A volcano plot (FDR = 0.01, s0 = 0.5) indicating inhibited farnesylated proteins (red) by tipifarnib. Ras is a protein group of HRas, Kras, and NRas.

We next sought to identify the farnesylated proteins responsive to inhibition. While immortalized cell lines are convenient for many laboratory experiments because they are easy to grow and can be passaged an unlimited number of times, their transformed nature can alter their physiology and render them less accurate as a disease model compared with primary cells. We first determined the set of prenylated proteins detectable in primary astrocytes using our probe labeling strategy in the presence of lovastatin. A total of 43 protein groups of prenylated proteins were identified, of which 33 individual prenylated proteins overlap (51%) with those identified from the immortalized astrocyte cell line (Fig. 6A). In contrast, 13 (20%) prenylated proteins were uniquely identified in the immortalized astrocytes and 19 (29%) were uniquely identified in the primary astrocytes. This suggests that there may be significant differences between the cell line and the primary cells and highlights how prenylomic profiling can be used to hone in on those differences. It is also noteworthy that the immortalized astrocytes used in this study were derived from the human APOE3 targeted replacement mice and thus some of the differences observed might result from the replacement of the APOE gene.

We have recently reported the farnesylated proteins responsive to perturbations in the farnesylation machinery in mouse brain with neuron-specific FTase deletion^47^. A total of 11 farnesylated proteins were identified in that study and are potentially implicated in the memory loss of the FTase-deficient mouse models. In this current study, we first evaluated the chemical inhibition of farnesylation using tipifarnib in the presence or absence of lovastatin through in-gel fluorescence analysis (Fig. 6B). Treatment with lovastatin enhanced probe labeling particularly in potentially farnesylated proteins near the 75 and 50 kDa regions (lane 3, indicated by arrows). Consistent with the aforementioned observations with cell lines, tipifarnib effectively suppresses labeling in these upper bands even in the presence of lovastatin (lane 5). Only minor differences in labeling near the 25 kDa region is apparent between tipifarnib treated samples (lanes 3 and 5) and their untreated counterparts (lanes 2 and 4). In order to identify these farnesylated proteins responsive to FTI inhibition, we performed our SPS-MS3 prenylomic approach in statin-treated primary astrocytes in the presence or absence of tipifarnib. We envisaged that since the C15AlkOPP probe can be incorporated by both FTase and GGTase-I, farnesylated proteins exhibiting the switch-like behavior would still be labeled under conditions where FTase is inhibited. Prenylomic analysis revealed that farnesylation substrates (15 protein groups, red) are inhibited to varying extents by tipifarnib (Fig. 6C). Six of these farnesylated proteins were identified as responsive to neuron-specific genetic deletion of FTase in mouse models^47^. Three of these proteins are above our stringent statistical threshold including Palm, Rheb and Lmnb1 with three more appearing slightly below the statistical threshold (Ston1, Dnaja1 and Ykt6). Ston1, a novel protein we identified in COS-7, displayed an appreciable level of inhibition, suggesting that this protein is truly a bona fide farnesylated protein. The assigned GGTase-I and Rab substrates are unaffected by FTI treatment. Of the other nine putative farnesylated proteins, statistically significant inhibition was not observed. Since the C15AlkOPP analogue used in these experiments is a substrate for both FTase and GGTase-I, the absence of inhibition is consistent with switch-like behavior suggesting that these proteins including the protein group Nras;Hras;Kras and Rras, can be alternatively geranylgeranylated in the presence of an FTI. A few other proteins including Dnaja4, Nap1I1 and Inpp5a show some limited inhibition that may be due to partial switch behavior or incomplete inhibition. Overall, these inhibition studies on primary astrocytes validate the potential farnesylation of Ston1 and support the switch like behavior of a number of proteins.

## Discussion

In this study, a chemical proteomic approach is described to identify the set of prenylated proteins in a variety of mammalian cells using a single alkyne-modified isoprenoid analogue. In terms of maximizing the number of prenylated proteins that can be identified, it is important to note that probe incorporation via metabolic labeling varies dramatically in different cell lines. Hence, careful selection of a cell line with maximal probe labeling is a prerequisite to maximize the number of prenylated proteins that can be identified. Here, we showed that lower labeling in cells not only impacts the extent of labeling of individual proteins but also reduces the total number of prenylated proteins that are enriched. Although prenylated proteins can be efficiently labeled by the C15AlkOPP probe in the presence of the endogenous isoprenoids, FPP and GGPP, lovastatin significantly enhances the incorporation efficiency as manifested by the increased fold enrichment and number of prenylated proteins identified. The dissociation constants (K_d_) across the prenyltransferase enzymes show tight binding with their corresponding isoprenoid substrates and are of comparable magnitude (FTase:FPP, 2.8 nM; GGTase-I:GGPP, 3 nM: RabGGTase:GGPP, 8 nM)^48–50^. However, it has previously been shown that the C15AlkOPP isoprenoid probe is a more efficient substrate for GGTase-I compared with FTase using comparable enzyme concentrations^51^. Therefore, it may functionally mimic GGPP better than FPP. Under physiological conditions, probe incorporation into farnesylated proteins is challenging because of competition with endogenous FPP. Suppression of the pool of native isoprenoids using statins may allow C15AlkOPP to more effectively compete for labeling of those more difficult to farnesylate with potentially lower native abundances. It is also important to note that the use of this probe does not necessarily impact the function of labeled substrates as previously described^52^. Therefore, this isoprenoid tagging approach may operate in viable cells with minimal perturbations in the physiological function of prenylated proteins.

The goal of defining the set of prenylated proteins in a mammalian system through different tagging strategies and proteomic approaches has been pursued for more than a decade^20,27,36,53^. Most early efforts through the use of enrichment strategies led to the identification of less than a few dozen prenylated proteins. Antibody-based detection through the use of aniline-functionalized isoprenoids were also reported but has led to only 22 prenylated proteins^19^. Over time, the number of proteins identified using enrichment methods has steadily risen as technology has improved. A recent report using a dual chemical probe strategy involving two separate isoprenoid analogues (used in their alcohol forms) to tag farnesylated and geranylgeranylated proteins in conjunction with SILAC and TMT analysis in EA.hy926, a human endothelial cell line, was employed for quantitative proteomic analysis^31^; a total of 80 prenylated proteins were reported, of which 64 were detected in the absence of a statin. Here, a simpler approach using a single analogue in its diphosphate form that is a substrate for all prenylating enzymes^27,28^ was employed; the use of the diphosphate form in these experiments may be particularly advantageous for physiological studies since it avoids potential toxicity caused by the presence of prenyl alcohols^54,55^. The resulting analysis yielded 78 and 59 protein groups in the presence and absence of lovastatin, respectively. This catalog of prenylated proteins is similar but not identical to the results obtained using the dual probe strategy showing extensive overlap in the identities of proteins profiled, as well as identifying unique sets of previously known and novel prenylated proteins. Four out of the seven novel proteins discovered in the earlier work^31^ were also identified in the analysis described here: CEP85, DCAF8, DPCD, and NAP1L4. Conversely, three additional novel prenylated proteins were identified here: PC4 and SFRS1-interacting protein PSIP1 (CaaX-box: CNLQ), Stonin-1 (CaaX-box: CITQ), and DHX32 (CaaX-box: CTLQ). Although the C-terminal peptides of these three proteins displayed multiple turnover activity for in vitro farnesylation in a previous study^56^, prenylation on their full-length forms has not been reported. Our results validate the farnesylation of these proteins, providing evidence of their prenylation in a cellular context. The differences in the prenylated proteins identified may be attributed to the differences in the cell line employed, labeling conditions, the enzymatic parameters associated with the probe used or the specific MS instrumentation employed for analysis. While extended C(X)_3_X-box containing proteins and Cxx peptides have been demonstrated to be in vitro prenylation substrates^5,6^, none of those proteins were found to be enriched in a statistically significant manner in our analysis. Several of these polypeptides do exist in mammalian proteomes, however their endogenous levels or efficiency of prenylation may be too low to be detected using the current chemical proteomic strategies. Finally, one cautionary feature related to these prenylomic experiments should be noted. In the strategy employed here, prenylated peptides are not directly observed in the LC–MS/MS analysis since enrichment relies on the alkyne functional group present in the isoprenoid analogue; lipidated peptides linked to biotin via click reaction remain bound to the neutravidin resin following tryptic digestion. Hence the identification of putative prenylated proteins is inferred from the detection of other non-prenylated peptides from the protein. The TMT-based method used here that compares enrichment in the presence and absence of alkyne probe allows most non-prenylated proteins to be excluded. However, this approach does not completely prevent proteins that associate with prenylated proteins from being enriched. There are limits to the stringency of washing conditions that can be employed since they must preserve sufficient protein structure to enable biotin-neutravidin interaction. In the analysis reported here, approximately 6% of the statistically enriched proteins were proteins with no apparent prenylation motif but were known to interact with prenylated proteins. While this number is small, it suggests that caution must be exercised in interpreting data from these experiments and that additional confirmatory experiments and/or data may be necessary to unambiguously identify novel prenylated proteins discovered using this metabolic labeling strategy. This underscores the need for better methods that allow for the enrichment of prenylated peptides that can be used to directly identify prenylated proteins.

Analyses presented here of the factors that influence the extent of enrichment of prenylated proteins in this chemical proteomic approach suggest that the catalytic efficiency of specific substrates for their cognate prenyltransferases does not correlate with their enrichment efficiency. A large number of prenylated proteins having CaaX-boxes with predicted higher farnesylation scores or prenylation efficiencies were not identified. This may be due to the low levels of expression of these proteins or to other regulatory mechanisms that involve chaperones that regulate the delivery of unprenylated proteins to their cognate prenyltransferase^57^. Currently, most of what is known concerning prenylation selectivity relies on data obtained from in vitro studies or computational experiments. Instead, it appears that the expression levels and native abundances of protein substrates have a greater impact on their detectability in these chemical proteomic experiments. In particular, CDC42 and RhoA have consistently emerged as two of the most enriched candidates across all cell lines studied here. Both of these contain efficiently prenylated C-terminal CaaX-box sequences^39^ and high native abundances or expression levels. Overall, protein levels are determined by a complex interplay of factors including mRNA transcript levels, transcript stability, translation efficiency and rate of protein degradation^58^. Given that metabolic labeling experiments provide a snapshot of the levels of many prenylated proteins in a single experiment, this approach holds great promise for investigating global changes that occur in specific disease states.

For chemical proteomic strategies, quantitative analysis is useful for evaluating the extent of probe incorporation into its cognate protein substrates. The evolution of orbitrap mass spectrometry instrumentation employed in proteomic studies led to improvements in sensitivity and accuracy of quantitation^59^. Consequently, more accurate quantitation can be achieved through the use of multinotch SPS-MS3 approach, where reporter ion interference that distorts reporter ion signals in MS2-based methods is obviated^60^. From the data reported here, using the multinotch approach with a more sensitive orbitrap instrument afforded a wider dynamic range of fold-change values compared to the MS2-based approach, allowing more accurate quantitation. Although there were some differences in the sets of prenylated proteins that were identified using the two different approaches, overall, the number of profiled prenylated proteins are comparable. Therefore, it appears that an MS2-level analysis of TMT-labeled peptides is generally sufficient to identify prenylated proteins derived from a chemical probe-based enrichment experiment. However, for applications where information regarding changes in the levels of specific prenylated proteins is important, the more accurate quantitation and wider dynamic range of the MS3-level approach make it a better choice.

As a prelude to future work directed at studying protein prenylation in brain-related diseases, we first sought to survey the prenylated proteins in several brain-derived cell lines including neurons, astrocytes, and microglia. Significant overlap (25 proteins, 42%) was found in the prenylated proteins identified in the three cell lines. Given that many of these are involved in cellular processes that are generally essential to cellular function, such as Ras signaling from Ras-related proteins, endocytosis, and chemokine signaling, this is not surprising (Supplementary Table S1). Intriguingly, some prenylated proteins are uniquely enriched in specific cell types. Eight proteins were observed exclusively in neurons, five were observed exclusively in astrocytes and three were observed exclusively in microglia, suggesting their unique roles in these cells. For example, among the eight proteins identified in neurons, Gng2 (G-protein gamma 2 subunit) is highly expressed there. Gng2 is a specific marker for the forebrain structure claustrum, which integrates multisensory information and promotes cognitive function^61,62^. Rac3, a member of the Rho family GTPases, is also abundantly expressed in neurons. Rac3 plays essential roles in neuronal development and related disorders^63^. The presence of the C-terminal prenylation site of Rac3 is specifically required for the maturation of neurons in culture. Hras, an exclusively farnesylated small GTPase, is modestly expressed in neurons; however, the role of Hras in regulating neuronal synaptic function is well established in vivo^64^. Overexpression or deletion of Hras significantly modulates neuronal function^65,66^. In cultured neurons, the activity and cellular localization of Hras, which depends on its farnesylation state, regulate nascent axonal growth^67^. Notably, another Ras protein, Rras, was also uniquely identified in neurons despite its minimal gene expression. Rras controls axon branching and neuronal polarity in cultured cortical/hippocampal neurons^68,69^. Among the five proteins uniquely identified in astrocytes, the small GTPase Rab34 is most highly expressed. Rab34 is a member of the secretory pathway and regulates phagolysosome biogenesis and cargo delivery^70,71^. These functions are critical for the normal role of astrocytes in the brain. Dysfunction of Rab34 leads to gliomas^72^. Among the three proteins uniquely identified in microglia, RhoG is most highly expressed. RhoG, a member of the Rho family of small GTPases, regulates neuroinflammation and the clearance of apoptotic cells, key functions of microglial cells in the brain^73,74^. In sum, our findings demonstrated the ability to track the levels of prenylated proteins in different brain cell types, many of which perform key regulatory roles within each cell type. The results will be highly useful in a variety of experiments seeking to explore the dysregulation of protein prenylation.

## Conclusion

The results presented here indicate that metabolic labeling of prenylated proteins in cultured cell lines can be accomplished using a single alkyne-modified isoprenoid analogue. The extent of labeling in each cell line differs, resulting in variations in enrichment and the number of prenylated proteins identified in chemical proteomic profiling. The native abundances of these proteins under steady-state conditions may dictate their ability to be enriched; however, the levels of prenyltransferase enzymes in different cell lines and their capability for probe uptake may also play crucial roles in determining the extent of prenylome labeling. Overall, this study shows that a common core of 19 prenylated proteins identified in 7 different cell types can be monitored using these types of experiments. This should provide important insights into the activity and regulation of signal transduction pathways in cells. In addition, prenylated proteins unique to specific cell types can also be identified and monitored using this approach. Eight proteins were observed exclusively in neurons, five were observed exclusively in astrocytes and three were observed exclusively in microglia, suggesting their unique roles in these cells. Finally, we report here that this powerful approach can be used to monitor protein prenylation in primary cells. Taken together, this powerful approach should be useful to unravel the role of prenylated proteins in myriad diseases.

## Methods

### Metabolic labeling in cultured cell lines and primary astrocytes

COS-7 and HeLa cells were generously provided by Dr. Elizabeth Wattenberg at the University of Minnesota. Mouse N2a neuroblastoma cells (ATCC CCL-131) were purchased from ATCC (Manassas, VA). Mouse BV2 microglial cells were generously provided by Dr. Hongwei Qin at the University of Alabama at Birmingham. The immortalized astrocytes were derived from the human APOE3 targeted replacement mice^75^ and were generously provided by Dr. G. William Rebeck at Georgetown University. Primary astrocytes were prepared and cultured from neonatal pups of wild-type C57BL6/J mice as described previously^76,77^. In brief, cortices and hippocampi from neonatal pups were dissected in cold HBSS, and enzymatically digested with 0.05% trypsin/EDTA for 20 min in 37 °C. Cells were triturated and passed through a 40 μm cell strainer. The cells were then centrifuged for 7 min at 1,000 rpm, and re-suspended in the culture medium DMEM Glutamax (Gibco, 10,569–010) containing 10% FBS (Gibco). After culturing for 2–4 weeks, microglial cells were removed by vigorously tapping the flask on the bench top and changing media. Remaining astrocytes were passaged and expanded in PDL-coated T75 flasks with DMEM containing 10% FBS and penicillin/streptomycin/amphotericin B (Antibiotic–Antimycotic, Gibco) and allowed to grow prior to plating into 100-mm dishes for metabolic labeling.

Cells (0.9 to 1.5 × 10^6^ seeding density) were grown in a 100-mm dishes containing 10 mL of DMEM (Gibco) media with 10% fetal bovine serum (Gibco) 1% penicillin–streptomycin (Gibco) one day prior to treatment. The media was removed and replaced with 5 mL of fresh media followed by pre-treatment with 10 μM lovastatin (Cayman Chemical) for 6 h under 5% CO_2_ at 37 °C. After pre-treatment, the media was either removed and replaced to remove the lovastatin or retained, followed by the addition of 10 μM C15AlkOPP or FPP and 3 μM tipifarnib when indicated. After 24 h of incubation, the cells were collected, pelleted through centrifugation and stored at -80 °C until further use.

### In-gel fluorescence labeling

In-gel fluorescence analysis of C15AlkOPP-treated cells was performed as previously described^29^. In brief, cell pellets were suspended in lysis buffer (10 mM Na_3_PO_4_ pH 7.4, 137 mM NaCl, 2.7 mM KCl, 2.4 μM PMSF, 200 units/nL benzonase nuclease (Sigma-Aldrich), protease inhibitor cocktail (1X PBS) and 1% SDS) and lysed by sonication on ice for 6–7 times with 2-s pulses at 10-s intervals. Protein concentrations were quantified using a BCA assay (Thermo Fisher Scientific) and 100 ug of the protein samples were subjected to click reaction with TAMRA-N_3_ (25 μM TAMRA-N_3_ (BroadPharm), 1 mM TCEP, 0.1 mM TBTA (Sigma-Aldrich), and 1 mM CuSO_4_) for 1 h at room temperature. Proteins were precipitated out using ProteoExtract precipitation kit (Calbiochem) and resuspended in 1X loading buffer containing 2% SDS, 10% glycerol, 0.02% bromophenol blue in 50 mM Tris–HCl pH 6.8. Labeled protein samples were resolved in 12% SDS-PAGE gels and detection for TAMRA fluorescence was performed using a Typhoon FLA 9500 (GE Healthcare). Gel images were processed in ImageJ.

### Enrichment of labeled prenylated proteins

Protein lysates (2 mg in 1 mL) from cells treated with lovastatin and C15AlkOPP or FPP were subjected to click reaction with biotin-N_3_ (100 μM biotin-N_3_ (BroadPharm), 1 mM TCEP, 0.1 mM TBTA, and 1 mM CuSO_4_) for 90 min at room temperature. Proteins were precipitated out using chloroform–methanol-water (1:4:3) to remove excess reagents and pelleted by centrifugation. Protein pellets were washed with methanol, redissolved in 1 mL of 1X PBS + 1% SDS and quantified using a BCA assay to normalize concentrations across all samples. Biotinylated samples (1 mg in 500 μL) were incubated with 100 μL (settled resin) of pre-washed Neutravidin agarose beads (Thermo Scientific) for 90 min. Beads were washed (1X PBS + 1% SDS (3x), 1X PBS (1x), 8 M urea in 50 mM TEAB (3x), and 50 mM TEAB (3x) with 1 mL per wash) and bound proteins were digested on-bead with 1.5 μg of sequencing grade trypsin (Promega Corp.) overnight at 37 °C. Peptides were collected by washing the beads with 100 μL 50 mM TEAB and dried by lyophilization.

### TMT-labeled sample preparation

Dried peptides were dissolved in 80 $\mu$L of 100 mM TEAB and quantified via BCA assay. Samples (10 μg) were supplemented with 150 fmol of internal standard (yeast ADH1, Waters) and subjected to labeling with TMT 6plex labeling (Thermo Fisher Scientific) following the manufacturer’s protocol. TMT-labeled samples were pooled, dried by lyophilization, and redissolved in 200 mM NH_4_HCO_2_, pH 10. Samples were fractionated under high pH reversed phase conditions on three layers of SDB-XC extraction disks (3 M, 1.07 mm × 0.50 mm i.d.) in 200 μL pipette tips into 60 μL volumes of 5,10, 15, 20, 22.5, 27.5, and 80% CH_3_CN in 200 mM NH_4_HCOO pH 10. The first two fractions were combined. The samples were dried and redissolved in 30 μL of 0.1% HCO_2_H in water and stored at − 80 °C until LC–MS analysis.

### Proteomic samples for quantifying native abundances

Lysates from COS-7 cells (40 $\mu$g, three biological replicates) were resolved using a 12% SDS-PAGE gel and stained with Coomassie Blue. The 20–30 kDa regions (~ 1 cm) were excised using a scalpel to evenly cut out bands across the lanes. Coomassie stain was removed by washing the gel pieces with 150 μL of 1:1 100 mM TEAB: CH_3_CN twice. Proteins in the gels were reduced with DTT (10 mM) at 56 °C for 1 h. Alkylation with iodoacetamide (55 mM) was performed in the dark for 30 min. Gel pieces were washed twice with the TEAB:CH_3_CN mixture and in-gel tryptic digestion was performed overnight at 37 °C with 10 ng/μL of trypsin. Tryptic peptides were collected and dried by lyophilization. Dried peptides were dissolved in 2% CH_3_CN in H_2_O with 0.1% HCO_2_H (Buffer A). Samples were supplemented with internal standard (yeast ADH1, 50 fmol, Waters) and loaded onto a pre-conditioned STAGE Tip (SDB-XC, 3 M). Samples were washed with Buffer A and eluted with 40% CH_3_CN in H_2_O with 0.1% HCO_2_H. Desalted peptides were dried and dissolved in 0.1% HCO_2_H in H_2_O. Proteomic samples were stored at -80 °C until LC/MS analysis.

### LC–MS data acquisition

The TMT-labeled peptides were resolved using a RSLC Ultimate 3000 nano-UHPLC (Dionex) with a reversed-phase column (75 μm i.d., 45 cm) packed in-house with ProntoSIL C18AQ 3 μm media at a flow rate of 300 nL/min. Each fraction from the high pH reversed-phase separation was subjected to a distinct gradient of solvent B (0.1% HCO_2_H in CH_3_CN) and solvent A (0.1% HCO_2_H in H_2_O) with amounts ranging between 7 and 34% of solvent B for 80 min and sprayed directly into the Orbitrap using a Nanospray Flex source (Thermo Fisher Scientific). For the multinotch SPS-MS3 approach, an Orbitrap Fusion Trihybrid (Thermo Fisher Scientific) mass spectrometer was used. MS1 scans were collected at 120,000 resolution in a 320–2,000 m*/z* range with a max injection time (IT) of 100 ms and an automatic gain control (AGC) target of 200,000. Subsequent data-dependent (top speed at 3 s) MS/MS scans were acquired using collision induced dissociation (CID) at a normalized collision energy (NCE) of 35% with a 1.3 m*/z* isolation window, a max IT of 100 ms, and an AGC target of 5,000. Dynamic exclusion was allowed for 60 s. Acquisition at MS3 was done by synchronously selecting the top 10 precursors for fragmentation by high-collisional energy dissociation (HCD) in the orbitrap with an NCE of 55% and a 2.5 m*/z* isolation window, 120 ms max IT, and 50,000 AGC target.

For label-free analysis of relative native abundances, each peptide sample was separated with 5–30% of Solvent B for 140 min. Data-dependent scans at MS1 were collected at 60,000 resolution over 320–2000 m*/z* range with AGC target of 500,000 and max IT of 50 ms. Dynamic exclusion was allowed for 90 s. HCD fragmentation was performed at NCE of 38% with 1.5 m*/z* isolation window, AGC of 50,000, and max IT of 200 ms.

The MS2-based quantitative proteomic analyses of TMT-labeled peptides were performed using an Orbitrap Elite instrument (Thermo Fisher Scientific) equipped with RSLC Ultimate 3000 nano-UHPLC and the same gradients used in SPS-MS3 approach were employed. A data-dependent acquisition was performed for MS1 scans under 60,000 resolution in a 350–1600 m*/z* range, with an AGC target of 1,000,000 and a max IT of 50 ms. Precursors were fragmented at MS2 using HCD at NCE of 40% with a 1.5 m*/z* isolation window, an AGC target of 50,000, and a max IT of 500 ms at 15,000 resolution.

### Proteomic data analysis

The raw files were searched using Andromeda embedded in MaxQuant (version 1.6.2.10) against the non-redundant human (UP000005640) or mouse (UP0000000589) databases from Uniprot (EMBL-EBI, April 2018 release). The peptides identified were based on full tryptic digestion with allowed missed cleavages of 3 with minimum peptide length of 7 residues. Fixed modifications were set for the TMT labels on both the N-terminal and lysine (229.163 Da) and variable modifications for methionine oxidation (15.995 Da) and N-term acetylation (42.010 Da). For label-free proteomic analysis, cysteine carbamidomethylation (57.021 Da) was used as a fixed modification instead. The unique + razor peptides were used for quantification. The default settings in the software for other parameters were used.

The resulting data were imported in Perseus (version 1.6.0.7) for filtering and statistical analysis. Proteins that were identified only by site, potential contaminant, or reversed were removed. The raw intensities were transformed to log2 values and proteins with less than 3 out of 6 values in each TMT channel were removed. Missing values were imputed based on a normal distribution. Reporter ion values were normalized by subtracting rows by means and columns by median. Statistical analysis was performed using two-sample *t*-tests with FDR = 0.01 and s0 = 0.5. Data were exported and processed in Microsoft Excel to generate volcano and fold-change correlation plots. For label-free proteomic analysis of the 20–30 kDa region proteins, LFQs were log2-transformed. Proteins identified in 2 out of 3 replicates were considered. Missing Log_2_(LFQ) values were imputed, normalized to the internal standard, yeast ADH1, and mean values were calculated. Prenylated proteins were extracted from the list of proteins identified on the basis of the presence of CaaX-box, existing annotations or validated in previous studies.

### Correlation analysis with published prenylation data

Previously reported prenylation data were used for correlation analyses. PrePS scores were obtained from http://mendel.imp.ac.at/PrePS/index.html and were adjusted by adding seven to obtain positive values. The FlexPepBind farnesylation scores were obtained from the complete list of 8000 CaaX-box peptide substrates published and provided by Dr. Ora Schueler-Furman^38^. The scores were adjusted by subtracting from three to obtain positive values. For both adjusted farnesylation scores, higher scores reflect greater propensity to farnesylation. The values for relative GGTase-I activity on peptide substrates were extracted from the thesis of Dr. Animesh V. Aditya from Purdue University. The percent prenylation on Rab protein substrates of RabGGTase determined in vitro were obtained from published data^40^. Kolmogorov–Smirnov (K-S) test was used to test for the normality of each variable. After assessing the normality of data, Spearman’s rank correlation test was used to measure the monotonic association between variables. Spearman’s rank correlation and regression analysis were performed on GraphPad Prism version 8.4.1. Graphs were formatted on Microsoft Excel.

## Supplementary Information

Supplementary Information 1.

Supplementary Information 2.
