# Supplementary material for: Metabolic labeling with an alkyne probe reveals similarities and differences in the prenylomes of several brain-derived cell lines and primary cells
Source: Sci Rep. 2021 Feb 23;11:4367. doi: 10.1038/s41598-021-83666-3 (PMC7902609; doi:10.1038/s41598-021-83666-3)
Supplement: Supplementary file 1 — Supplementary Information 1. [file 41598_2021_83666_MOESM1_ESM.pdf]

Supporting Information

Metabolic labeling with an alkyne probe reveals similarities and differences in the prenylomes of several brain-derived cell lines and primary cells

Kiall F. Suazo[†], Angela Jeong[‡], Mina Ahmadi[†], Caroline Brown[†], Wenhui Qu[§], Ling Li[‡§], Mark D. Distefano[†]*

[†]*Department of Chemistry, University of Minnesota, Minneapolis, MN 55455 USA*

[‡]*Department of Experimental and Clinical Pharmacology, University of Minnesota, Minneapolis, MN 55455 USA*

[§]*Department of Neuroscience, University of Minnesota, Minneapolis, MN 55455, USA*

*Corresponding Author: Mark Distefano, diste001@umn.edu

Table of Figures and Tables

Figure S1. Exploring the factors affecting prenylation efficiency. 1

Figure S2. Comparison of native abundance of proteins near the 25 kDa region of samples from COS-7 in the presence or absence of lovastatin. 2

Figure S3. The prenylated proteins in HeLa were identified in COS-7................................. 3

Figure S4. Volcano plots generated from the prenylomic analysis in neurons, astrocytes, microglia, and primary astrocytes.. 4

Figure S6. Dependence of enrichment fold-changes on mRNA levels of prenylated proteins identified from brain-derived cell lines and primary astrocytes 6

Figure S7. Response to FT inhibition using tipifarnib in various cell lines........................... 7

Table S1. Pathway analysis on the prenylated proteins identified shared by all three brain cell lines...8

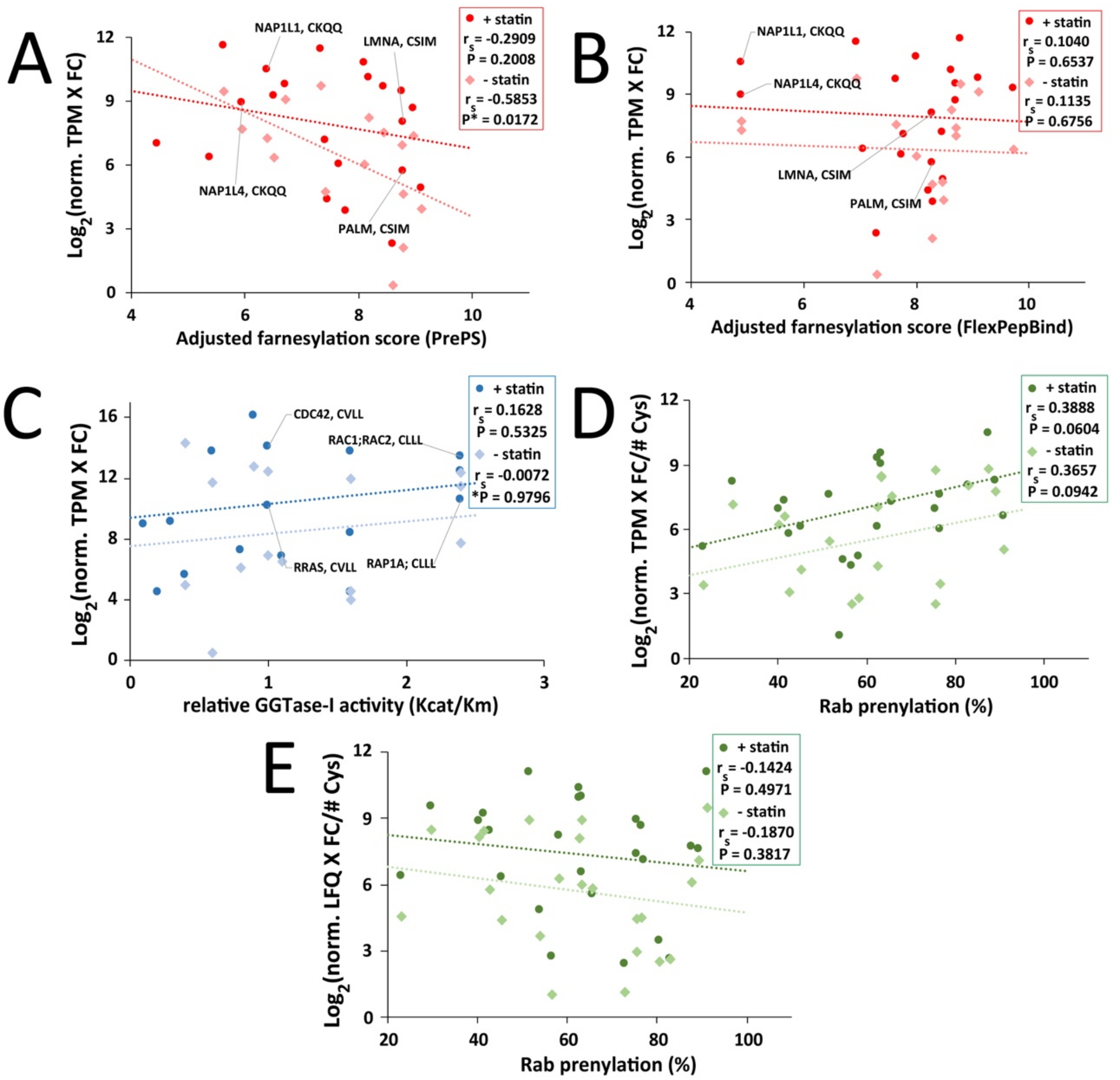

Figure S1. Exploring the factors affecting prenylation efficiency. Dependence of normalized enrichment fold-changes on farnesylation scores from PrePS **(A)** and FlexPepBind **(B)**, GGTase activity using peptide substrates **(C)** and percent prenylation of Rab proteins *in vitro* **(D)** and **(E).** No significant correlation was observed between enrichment fold-changes and these parameters. The mRNA levels (TPM) were used to normalize the enrichment fold changes in A-D while the native abundances (LFQ) were used for E. The number of prenylatable cysteines in Rab proteins were also accounted for in the normalization.

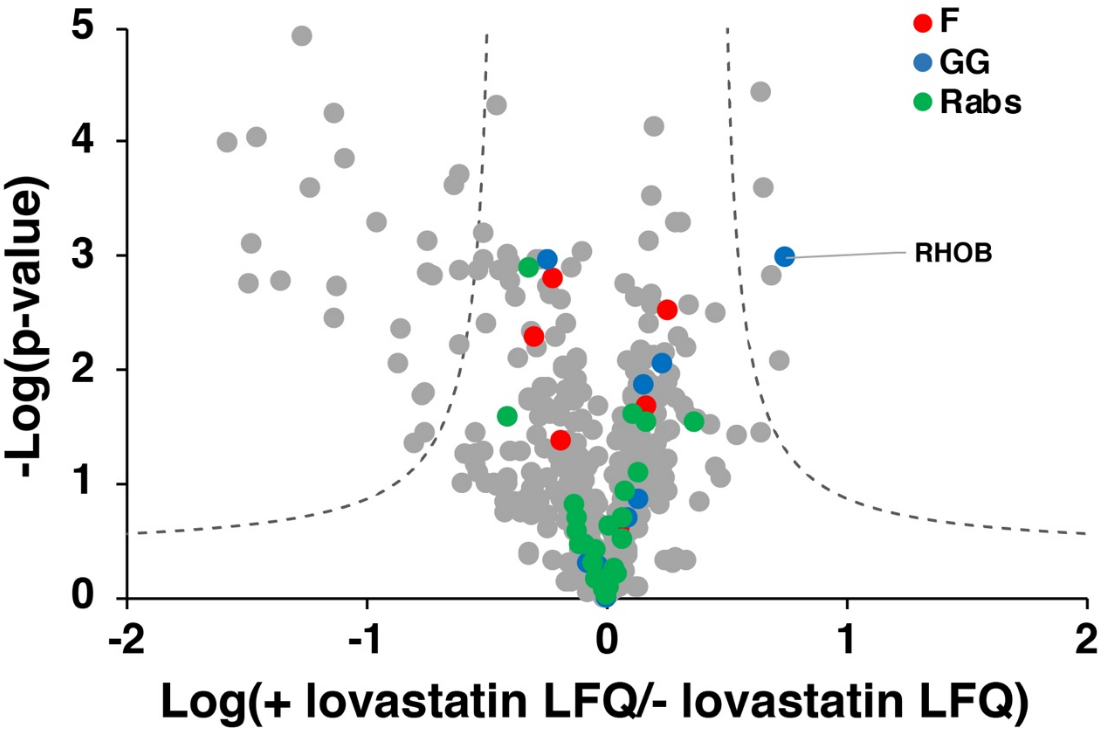

Figure S2. Comparison of native abundance of proteins near the 25 kDa region of samples from COS-7 in the presence or absence of lovastatin.

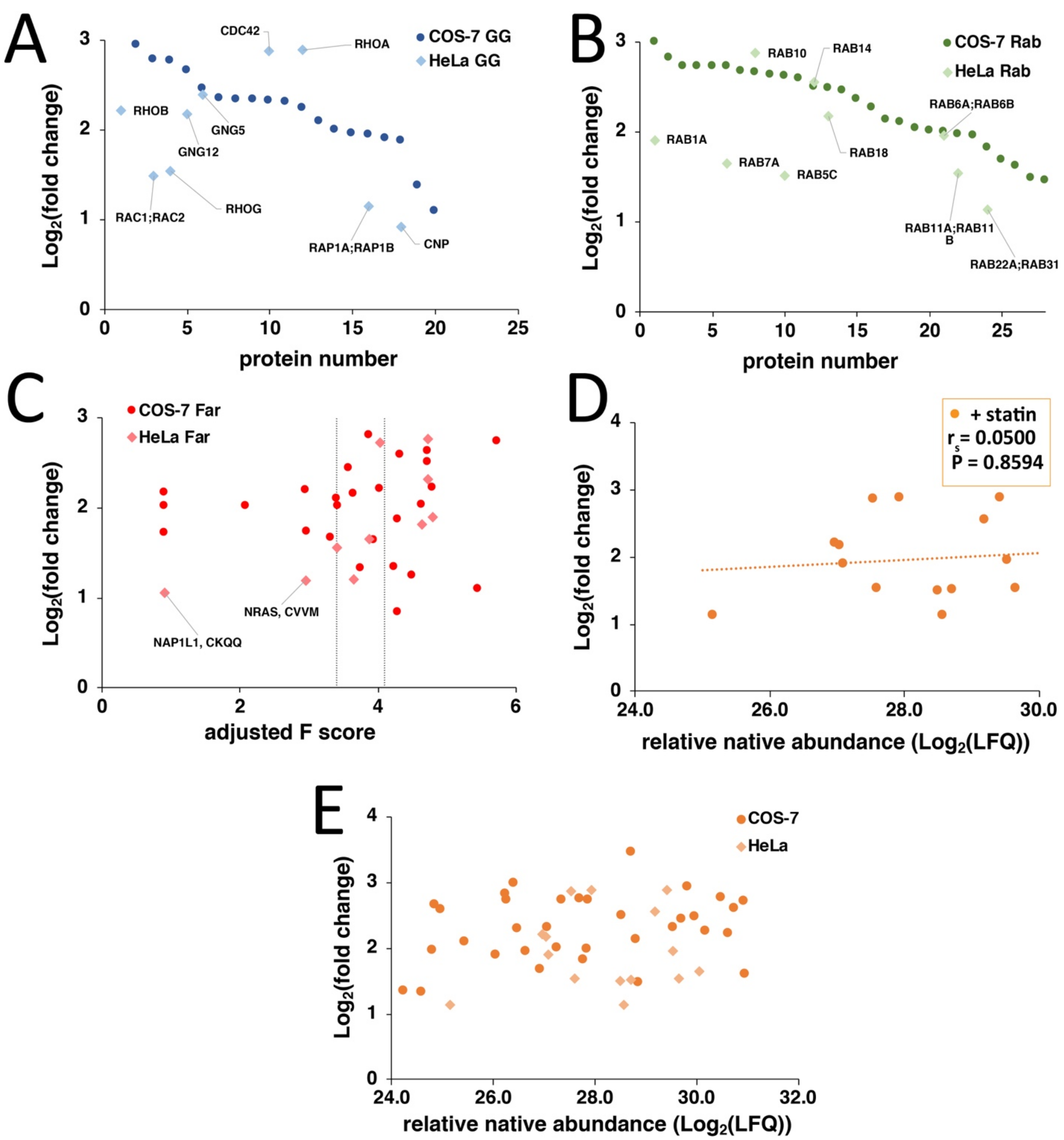

Figure S3. The prenylated proteins in HeLa were identified in COS-7. **(A)** GGtase-I substrates and **(B)** Rab proteins identified in HeLa were also identified in COS-7 in a wide dynamic range of enrichment fold-changes. **(C)** The farnesylated proteins identified in HeLa were also identified in COS-7 with a wide range of assigned FlexPepBind farnesylation scores. Cut-offs shown in vertical lines for low, medium and highly predicted to be substrates of farnesylation. **(D)** Correlation analysis between the enrichment fold-change and relative native abundances of GGTase-I substrates and Rab proteins identified from MS2-based analysis on HeLa in the presence of statin. No significant Spearman's correlation was observed, **(E)** Most small GTPase and Rab proteins identified in HeLa are those that have relatively higher abundance in both HeLa and COS-7.

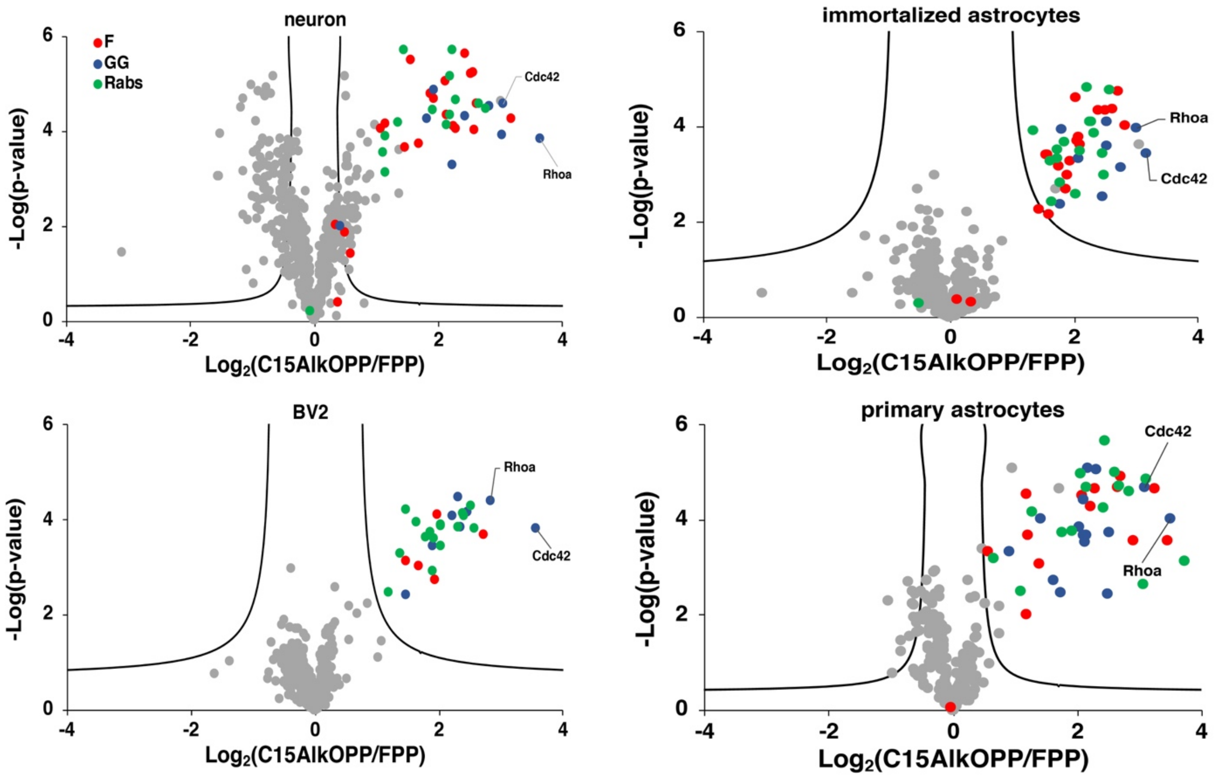

Figure S4. Volcano plots generated from the prenylomic analysis in neurons, astrocytes, microglia, and primary astrocytes. Stronger background labeling was observed in neurons. Statistical parameters: FDR = 0.01, s0 = 0.5.

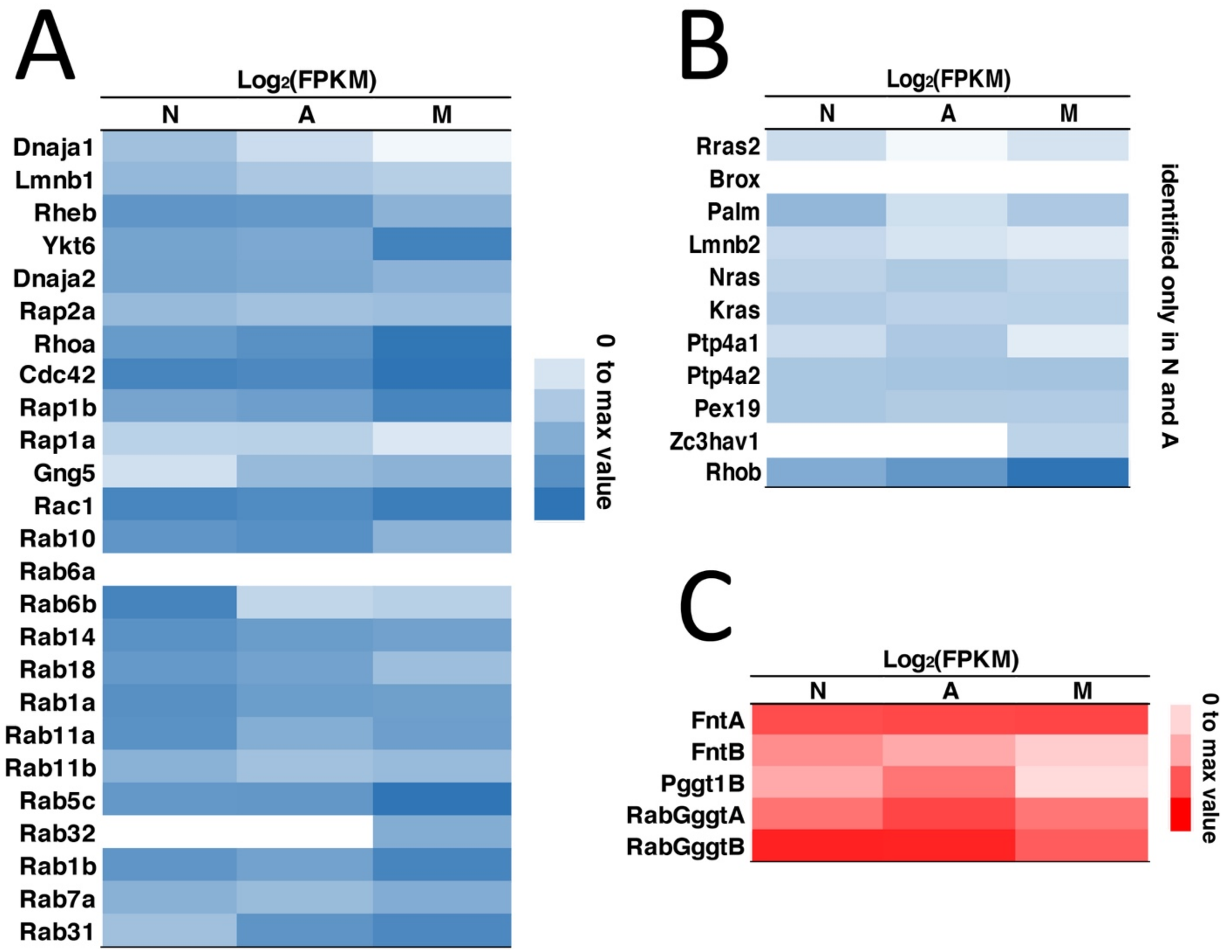

Figure S5. Relative mRNA expression levels of prenylated proteins in brain cells. (A) Relative mRNA expression levels of the identified prenylated proteins shared by neuronal cells (N), astrocytes (A), and microglial cells (M). **(B)** Relative expression levels of prenylated proteins statistically enriched and identified in neurons and astrocytes but not in microglia. **(C)** Relative expression of the prenyltransferase enzymes.

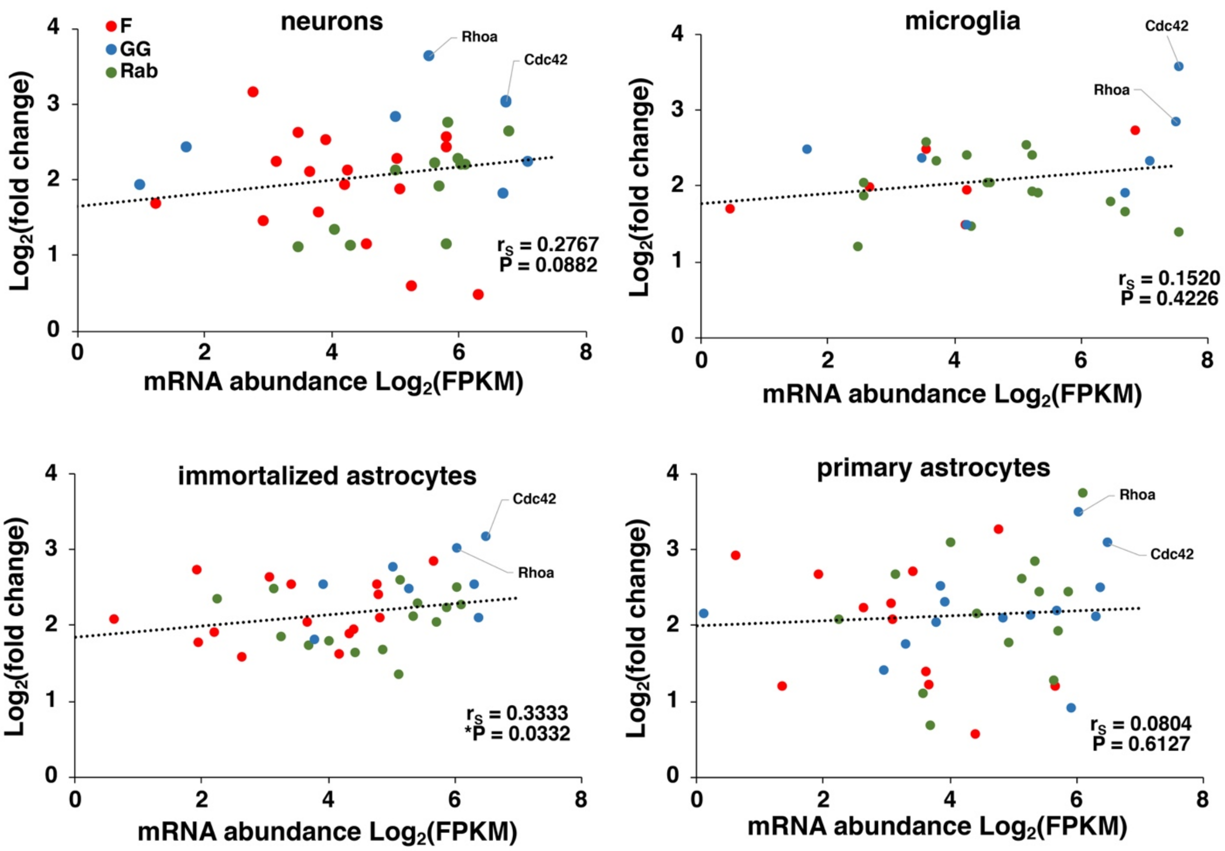

Figure S6. Dependence of enrichment fold-changes on mRNA levels of prenylated proteins identified from brain-derived cell lines and primary astrocytes. Cdc42 and RhoA were consistently identified as two of the most enriched proteins also display high levels of expression.

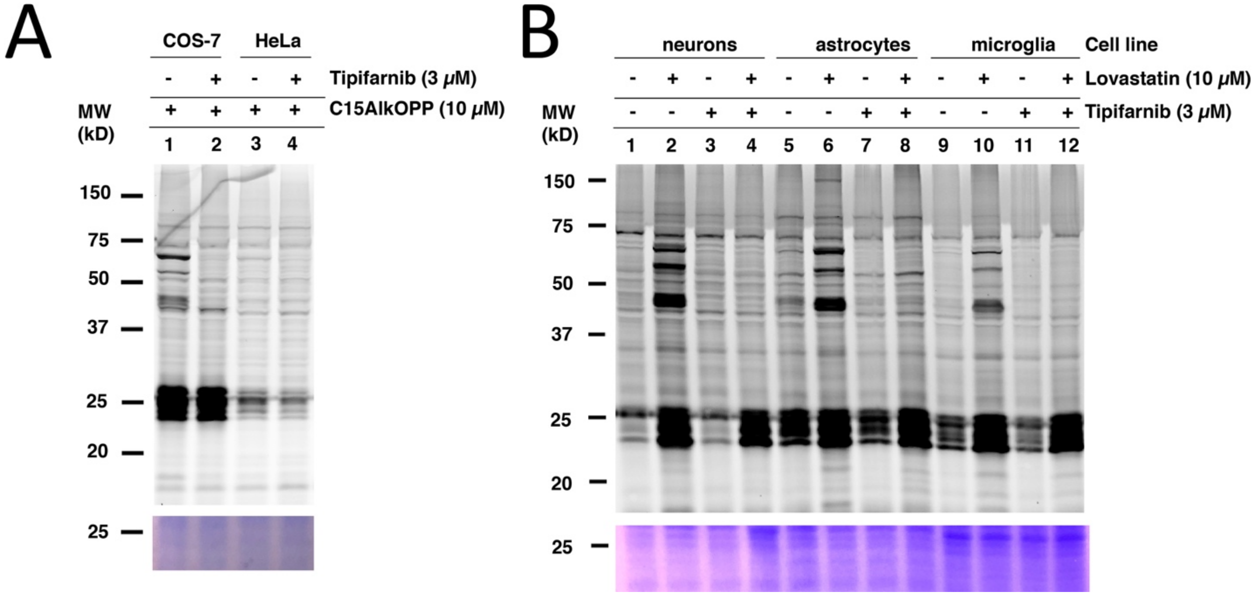

Figure S7. Response to FT inhibition using tipifarnib in various cell lines. **(A)** Tipifarnib treatment in COS-7 and HeLa cells. **(B)** Tipifarnib treatment in brain-derived cell lines in the presence or absence of lovastatin
.

Table S1. Pathway analysis on the prenylated proteins identified shared by all three brain cell lines. Pathways are ranked based on p-values and sourced from KEGG Mouse 2019 using Enrichr analysis tool.[1] The top ten pathways were selected.

Index	Term	P-value	Adjusted P-value	Odds Ratio	Combined Score
1	Ras signaling pathway	1.07E-08	3.24E-06	24.03	441.10
2	Endocytosis	2.88E-08	4.36E-06	20.82	361.47
3	Chemokine signaling pathway	1.28E-07	1.30E-05	24.37	386.67
4	Pancreatic secretion	1.77E-07	1.34E-05	38.10	592.26
5	Leukocyte transendothelial migration	2.79E-07	1.69E-05	34.78	524.93
6	Neurotrophin signaling pathway	3.60E-07	1.82E-05	33.06	490.52
7	Renal cell carcinoma	1.46E-06	6.34E-05	47.06	632.18
8	Focal adhesion	4.19E-06	1.59E-04	20.10	248.90
9	Rap1 signaling pathway	5.32E-06	1.79E-04	19.14	232.41
10	AMPK signaling pathway	1.71E-05	5.19E-04	25.40	278.70

REFERENCES

1. Kuleshov, M. V *et al.* Enrichr: a comprehensive gene set enrichment analysis web server 2016 update. *Nucleic Acids Res.* **44**, W90–W97 (2016).
